# Interfacial Engineered Vanadium Oxide Nanoheterostructures Synchronizing High-Energy and Long-Term Potassium-Ion Storage

**DOI:** 10.1021/acsnano.1c09935

**Published:** 2022-01-11

**Authors:** Xiaoxiao Kuai, Ke Li, Jianmei Chen, Hao Wang, Junyi Yao, Chao-Lung Chiang, Tingting Liu, Hanzhang Ye, Jianqing Zhao, Yan-Gu Lin, Labao Zhang, Valeria Nicolosi, Lijun Gao

**Affiliations:** † Soochow Institute for Energy and Materials Innovations & Key Laboratory of Advanced Carbon Materials and Wearable Energy Technologies of Jiangsu Province, College of Energy, Soochow University, Suzhou 215006, China; ‡ School of Chemistry, Centre for Research on Adaptive Nanostructures and Nanodevices (CRANN) & Advanced Materials and BioEngineering Research (AMBER), Trinity College Dublin, Dublin, Dublin 2, Ireland; § College of Electronic and Optical Engineering & College of Microelectronics, Nanjing University of Posts and Telecommunications, Nanjing, 210023, P. R. China; ⊥ Research Institute of Superconductor Electronics, School of Electronic Science and Engineering, Nanjing University, Nanjing 210023, China; ∥ National Synchrotron Radiation Research Center, Hsinchu 30076, Taiwan, R.O.C; ¶ School of Environmental Science and Engineering& Jiangsu Key Laboratory of Environmental Science and Engineering, Suzhou University of Science and Technology, Suzhou 215001, China

**Keywords:** 3D structure, energy storage, battery, capacitor, potassium-ion

## Abstract

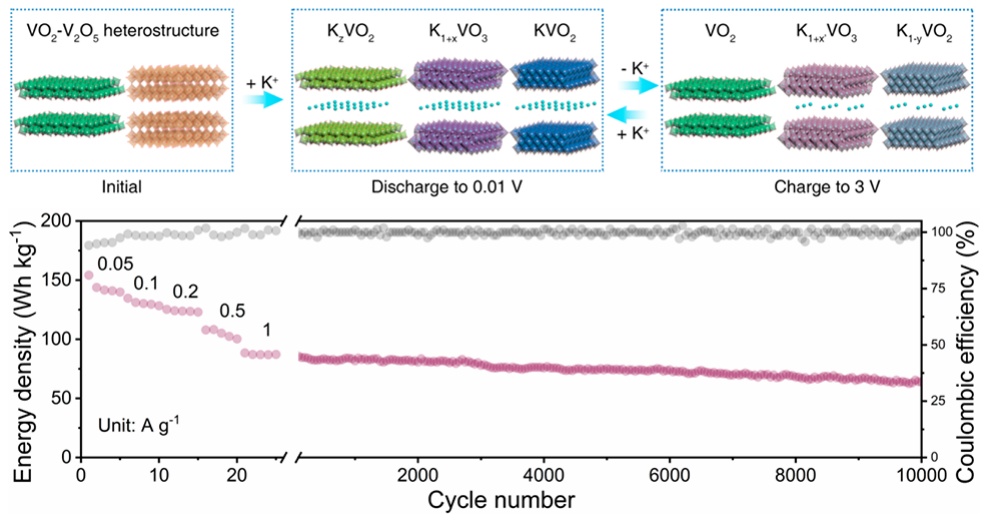

Potassium ion hybrid capacitors (KICs) have drawn tremendous attention for large-scale energy storage applications because of their high energy and power densities and the abundance of potassium sources. However, achieving KICs with high capacity and long lifespan remains challenging because the large size of potassium ions causes sluggish kinetics and fast structural pulverization of electrodes. Here, we report a composite anode of VO_2_–V_2_O_5_ nanoheterostructures captured by a 3D N-doped carbon network (VO_2_–V_2_O_5_/NC) that exhibits a reversible capacity of 252 mAh g^–1^ at 1 A g^–1^ over 1600 cycles and a rate performance with 108 mAh g^–1^ at 10 A g^–1^. Quantitative kinetics analyses demonstrate that such great rate capability and cyclability are enabled by the capacitive-dominated potassium storage mechanism in the interfacial engineered VO_2_–V_2_O_5_ nanoheterostructures. The further fabricated full KIC cell consisting of a VO_2_–V_2_O_5_/NC anode and an active carbon cathode delivers a high operating voltage window of 4.0 V and energy and power densities up to 154 Wh kg^–1^ and 10 000 W kg^–1^, respectively, surpassing most state-of-the-art KICs.

## Introduction

Large-scale and low-cost energy storage systems are becoming increasingly important in our society because of their ability to power industrial manufacturing and electric vehicles, store renewable energies (e.g., solar, wind, and hydropower energies), and balance power grids.^1^ Lithium-ion batteries and electrochemical capacitors are the two typical energy storage devices,^1−5^ and capacitors possess much higher power densities and longer lifespans than batteries. By constructing hybrid capacitors with battery-type anodes and capacitive-type cathodes, the limited energy density of capacitors can be prominently enhanced, reaching or even surpassing the levels of traditional batteries, meanwhile maintaining high power density outputs.^6−8^ Nonetheless, the rise in the cost of lithium and the rapid depletion of lithium sources prevent the large-scale deployment of lithium-ion hybrid capacitors (LICs).

As a potential alternative, potassium reserves in the Earth’s crust are nearly 1000 times greater than lithium sources. Meanwhile, it also features a low reduction potential of −2.93 V vs SHE, similar to the value of lithium (−3.04 V), promising for designing KICs with electrochemical performance comparable to LICs.^9,10^ However, most of the reported KICs suffer from moderate energy density and electrochemical stability,^11−28^ due to the large ionic radius of K^+^ (1.38 Å). In KICs, only partial active sites of usual electrode materials can be occupied by K^+^, and the insertion/extraction of large K^+^ also leads to significant and irreversible electrode pulverization during charge–discharge processes. As a result, developing electrode materials with high capacities and enhanced structural stability throughout long-term cycling is critical to the application of KICs.^29^

Among various potassium-ion storage electrode materials, vanadium oxides with wide diversity (e.g., VO_2_, V_2_O_3_, V_2_O_5_, and V_3_O_7_), high theoretical capacities,^30−33^ and low cost have attracted great research interests,^34^ and some progress has been achieved.^35−37^ For instance, a surface-amorphized VO_2_ anode was reported to deliver a capacity of 177.1 mAh g^–1^ after 500 cycles at 0.5 A g^–1^.^35^ More recently, heterojunctions are emerging as promising electrode material candidates because of their striking interface effects and the intrinsic generation of a built-in electric field at the heterointerface induced by work function difference. These features can remarkably alleviate interfacial stress and boost reaction kinetics and electron/ion transport, thus leading to enhanced electrochemical performance,^38−40^ which may also suit vanadium oxides.

Herein, VO_2_–V_2_O_5_ heterostructures with an ultrasmall size of ∼5 nm encapsulated on 3D N-doped carbon networks are developed through a self-template strategy. Benefiting from the ultrasmall size of VO_2_–V_2_O_5_ heterojunctions, favorable interfacial effect, high-conductive 3D carbon network, and distinctive K^+^ storage mechanism, VO_2_–V_2_O_5_/NC demonstrates great performance for potassium-ion storage, such as a reversible capacity of 501 mAh g^–1^ at 0.1 A g^–1^ after 200 cycles and a rate capability with 108 mAh g^–1^ being retained at 10 A g^–1^ in a K-ion half battery. Moreover, the constructed KICs comprising VO_2_–V_2_O_5_/NC anodes and commercial activated carbon (AC) cathodes provide a maximum energy density and power density up to 154 Wh kg^–1^ and 10 000 W kg^–1^, respectively, as well as exceptional long-term stability during 10 000 cycles.

## Results and Discussion

### Synthesis and Characterization of VO_2_–V_2_O_5_/NC

**Figure 1:**
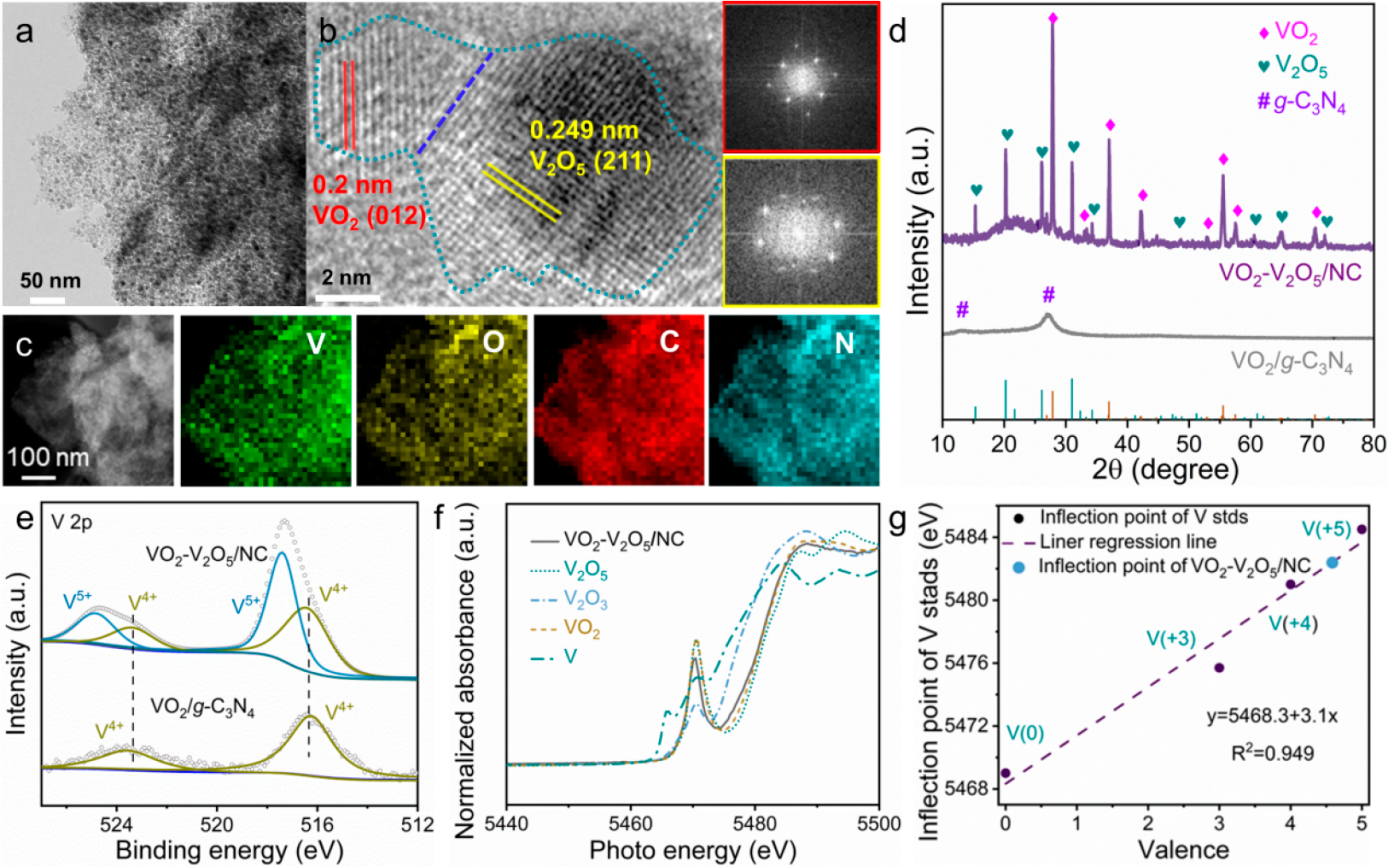
Characterization of the prepared VO_2_–V_2_O_5_/NC sample. (a) TEM image of VO_2_–V_2_O_5_/NC, where the blue dashed line shows the interface of the VO_2_–V_2_O_5_ heterostructure. (b) HRTEM image of a VO_2_–V_2_O_5_ nanoheterostructure anchored on N-doped carbon, and the corresponding fast Fourier transformation patterns of VO_2_ (red) and V_2_O_5_ (yellow). (c) HAADF-STEM image and the corresponding elemental mapping images of V, N, C, and O in VO_2_–V_2_O_5_/NC. (d) XRD patterns of VO_2_–V_2_O_5_/NC and VO_2_/*g*-C_3_N_4_. (e) High-resolution XPS spectra of V 2p of VO_2_–V_2_O_5_/NC and VO_2_/*g*-C_3_N_4_. (f) V K-edge XANES spectra of V, VO_2_, V_2_O_3_, V_2_O_5_, and VO_2_–V_2_O_5_/NC. (g) Linear regressions of vanadium valence derived from relevant standards for VO_2_–V_2_O_5_/NC.

VO_2_–V_2_O_5_/NC was synthesized via a self-template strategy (Figure S1). The mixture precursor of oxalic acid, dicyandiamide, and ammonium vanadate was first annealed at 550 °C under a N_2_ atmosphere. During this process, dicyandiamide was transformed into intermediate graphitic carbon nitride (*g*-C_3_N_4_) bulk, while ammonium vanadate was reduced into VO_2_,^41^ resulting in a VO_2_/*g*-C_3_N_4_ composite (Figure S2). Followed by annealing at 800 °C in N_2_ flow (VN/NC, Figure S3) and partially oxidizing at 280 °C in air, the final target product VO_2_–V_2_O_5_/NC was obtained (Figure 1b,d and Figure S4). Generally, dicyandiamide will be transformed into *g*-C_3_N_4_ at ∼530 °C and then fully decomposed at temperatures above 700 °C.^42^ However, in our case, because of the electrostatic interaction between the protonation of lone-pair electrons of pyridinic N in *g*-C_3_N_4_ and (VO)_2_(C_2_O_4_)_3_^2–^ pieces,^43^*g*-C_3_N_4_ was ultimately carbonized into 3D N-doped carbon networks at 800 °C (Figure S5), together with the formation of VN from VO_2_ (Figure S6). Meanwhile, in the later pyrolysis process, the as-formed 3D carbon networks maintained and protected VO_2_–V_2_O_5_ from irreversible fusion and aggregation, which was distinctly different from the compact and bulky VO_2_/*g*-C_3_N_4_ (Figure S7) and *g*-C_3_N_4_.^44^ As confirmed by the transmission electron microscopy (TEM) and scanning electron microscopy (SEM) images of VO_2_–V_2_O_5_/NC in Figure 1a and Figure S8a, a porous carbon framework is homogeneously implanted with massive VO_2_–V_2_O_5_ nanocrystals (size: ∼5 nm). The lattice fringes with interlayer spacings of 0.249 and 0.2 nm in the high-resolution TEM (HRTEM) image (Figure 1b) correspond to the (012) and (211) planes of VO_2_ and V_2_O_5_, respectively. High-angle annular dark-field scanning transmission electron microscopy (HAADF-STEM) and corresponding elemental mapping images (Figure 1c) also confirm the uniform dispersion of ultrasmall VO_2_–V_2_O_5_ nanoheterostructures. To further investigate the microstructure of VO_2_–V_2_O_5_/NC, N_2_ adsorption/desorption measurements were employed. As presented in Figure S9, a type-II isotherm with a hysteresis loop and a narrow pore distribution centered at 3 nm are observed in VO_2_–V_2_O_5_/NC, indicating its mesoporous structure.^45^ In addition, the Brunauer–Emmett–Teller (BET) surface area of VO_2_–V_2_O_5_/NC was measured to be 378.5 m^2^ g^–1^, much higher than that of VO_2_/*g*-C_3_N_4_ (2.1 m^2^ g^–1^) (Table S1). Notably, the mesoporous structure and large surface area will facilitate electrolyte ion diffusion and charge transfer, thus enhancing the electrochemical performance of VO_2_–V_2_O_5_/NC.

X-ray-based measurements were employed to finely explore the crystal structure, composition, phase, and valence information on the obtained samples. In the XRD diffractogram (Figure 1d), the two peaks corresponding to *g*-C_3_N_4_ in VO_2_/*g*-C_3_N_4_ confirm the formation of *g*-C_3_N_4_ during the annealing process.^46^ In contrast, the obtained VO_2_–V_2_O_5_/NC shows the highest crystallinity with a series of sharp peaks, which match well with the simulated VO_2_ (PDF#44-0252) and V_2_O_5_ (PDF#41-1426) patterns. The survey X-ray photoelectron spectroscopy (XPS) spectrum also reveals the presence of V, O, C, and N elements in VO_2_–V_2_O_5_/NC (Figure S10a). The N 1s XPS spectrum of VO_2_–V_2_O_5_/NC (Figure S10b) can be deconvolved into pyridinic N (398.8 eV), pyrrolic N (400 eV), and graphitic N (401.2 eV). Notably, pyridinic N and pyrrolic N in N-doped carbon-based materials have been demonstrated to contribute some capacities.^47,48^ For the high-resolution V 2p spectrum of VO_2_–V_2_O_5_/NC (Figure 1e), the two group peaks corresponded to V 2p_1/2_ and 2p_3/2_, clearly demonstrating the existence of V^4+^ and V^5+^ in this composite. Figure 1f shows the vanadium K-edge X-ray absorption near edge structure (XANES) spectra of relevant vanadium standards (VO_2_, V_2_O_3_, V_2_O_5_) and VO_2_–V_2_O_5_/NC. These samples with various vanadium valences were used as standards to estimate the oxidation states of vanadium species by linear fitting the inflection point position in the edge absorption region.^49^ Once again, the existence of both V^4+^ and V^5+^ in VO_2_–V_2_O_5_/NC is confirmed, and the average valence of vanadium is derived to be 4.58 (42 wt % VO_2_ and 58 wt % V_2_O_5_) via the vanadium oxide-derived linear regression line (Figure 1g). Moreover, on the basis of the thermogravimetric analysis (Figure S11), the mass percentage of VO_2_–V_2_O_5_ in VO_2_–V_2_O_5_/NC composite is 39.8%.

### Electrochemical Measurements of VO_2_–V_2_O_5_/NC Electrode

**Figure 2:**
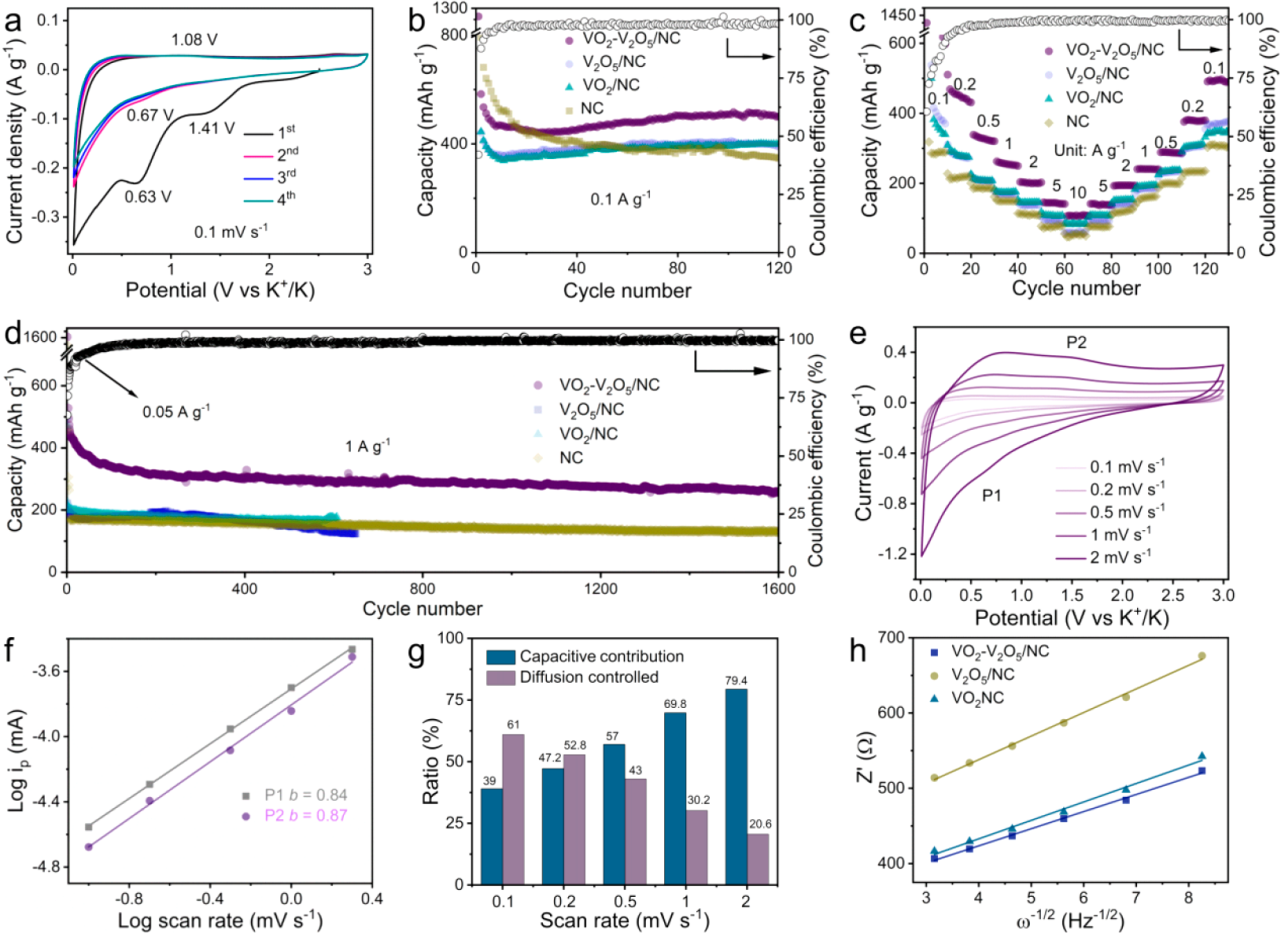
Electrochemical performance of VO_2_–V_2_O_5_/NC in a half-cell system. (a) CV curves of the VO_2_–V_2_O_5_/NC electrode at a scan rate of 0.1 mV s^–1^. (b) Electrochemical performance of electrodes at 0.1 A g^–1^. (c) Rate capabilities of electrodes at different current densities. (d) Long cycle performance of electrodes at 1 A g^–1^. (e) CV curves of VO_2_–V_2_O_5_/NC at different scan rates. (f) *b*-values of the VO_2_–V_2_O_5_/NC electrode. (g) Capacitive and diffusion-controlled contribution ratios in the VO_2_–V_2_O_5_/NC electrode at different scan rates. (h) Linear fit of Z′ against ω^–1/2^ for VO_2_–V_2_O_5_/NC, VO_2_/NC, and V_2_O_5_/NC electrodes, in which the values are derived from Figure S15a.

Figure 2a shows the first four cyclic voltammetry (CV) cycles of VO_2_–V_2_O_5_/NC at a scan rate of 0.1 mV s^–1^. Two reduction peaks centered at 1.41 and 0.63 V are observed in the first cathodic cycle, which can be ascribed to the initial insertion of K^+^ into VO_2_ and V_2_O_5_ lattices (Figure S12b, 13b), accompanied by the unavoidable formation of SEI film and irreversible phase transition reactions,^23,24^ and the subsequent reversible potassiation/depotassiation is corroborated by the stable redox peaks at 1.08/0.67 V in the following cycles. When cycling at a low current density of 0.1 A g^–1^, VO_2_/NC and V_2_O_5_/NC electrodes both show some capacity decays, while VO_2_–V_2_O_5_/NC maintains a high capacity 501 mAh g^–1^ after 120 cycles, which is substantially greater than VO_2_/NC (399 mAh g^–1^), V_2_O_5_/NC (388.9 mAh g^–1^), and NC (346.5 mAh g^–1^) (Figure 2b), indicating the enhanced structural stability and higher electrochemical activity of the VO_2_–V_2_O_5_/NC electrode. Moreover, the capacity contributions of the VO_2_–V_2_O_5_ heterostructure and NC in VO_2_–V_2_O_5_/NC electrode at 0.1 A g^–1^ are calculated to be 59.1% and 40.9%, respectively, according to their mass contents determined by TGA (Figure S6). The increasing capacity of VO_2_–V_2_O_5_/NC during cycling mainly derives from the gradual activation process of electrode materials, which happens in other metal oxide materials as well.^50^ The rate capabilities of these electrodes and the charge/discharge behaviors at different current densities of the VO_2_–V_2_O_5_/NC electrode were further investigated, as displayed in Figure 2c and Figure S14. The VO_2_–V_2_O_5_/NC electrode delivers highly reversible capacities of 510, 431, 321, 259, 202, 142, and 108 mAh g^–1^ at current densities of 0.1, 0.2, 0.5, 1, 2, 5, and 10 A g^–1^, respectively, certainly surpassing VO_2_/NC, V_2_O_5_/NC, and NC. Moreover, when the current density goes back to 0.1 A g^–1^, the specific capacity of VO_2_–V_2_O_5_/NC almost recovers (498 mAh g^–1^). Figure 2d shows the long-term cycling performance of different electrodes at a current density of 1 A g^–1^. Remarkably, the VO_2_–V_2_O_5_/NC electrode exhibits a high reversible capacity of 256 mAh g^–1^ with 100% Coulombic efficiency (CE) even after 1600 cycles, much better than VO_2_/NC, V_2_O_5_/NC and most state-of-the-art K^+^ storage electrodes (Table S2). The improved cycle stability of VO_2_–V_2_O_5_/NC is expected to be due to the strong stress migration ability at the interface of ultrasmall VO_2_–V_2_O_5_ heterostructures toward the volume fluctuation and the effective strain release caused by the extremely small nanocrystalline during charge–discharge processes;^26−28^ meanwhile, the N-doped carbon network may also help suppress particle pulverization.^50^

To interpret the potassium ion storage kinetics in VO_2_–V_2_O_5_/NC electrode, CV tests at different scan rates were conducted (Figure 2e and Figure S16). The peak current (i) and scan rate (*v*) follow this formula: *i* = *av*^*b*^, where *a* and *b* are coefficients: *b* = 0.5 means a semi-infinite diffusion control reaction, while *b* = 1 corresponds to a capacitive process.^51^ As shown in Figure 2f, the calculated *b*-values of the two redox peaks P1 and P2 are 0.84 and 0.87, respectively. They are both close to the value of 1, revealing that the charge storage processes in VO_2_–V_2_O_5_/NC are predominantly capacitive^52−54^ (*b* values of VO_2_/NC and V_2_O_5_/NC are shown in Figure S17). Moreover, the contributions from surface capacitive effects and diffusion-controlled reactions in the VO_2_–V_2_O_5_/NC electrode were measured using a voltammetric sweep rate-dependent analysis:^55^
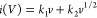
1where *k*_1_ and *k*_2_ are appropriate values. At a given potential, the current (*i*) is composed of surface capacitive effects (*k*_1_*v*) and diffusion-controlled processes (*k*_2_*v*^1/2^). As shown in Figure S18, the surface capacitive effect is the primary contributor at 2 mV s^–1^, which accounts for 79.4% of the total capacitance of the VO_2_–V_2_O_5_/NC electrode. Figure 2g summarizes the capacitive contribution at different scan rates. The capacitive contribution in VO_2_–V_2_O_5_/NC increases gradually from 39% at 0.1 mV s^–1^ to 74.9% at 2 mV s^–1^, indicating that capacitive processes are dominant at high scan rates, which is in line with the great rate capabilities of VO_2_–V_2_O_5_/NC.

The electrochemical process differences between VO_2_–V_2_O_5_/NC, VO_2_/NC, and V_2_O_5_/NC (Figure S15a) were also evaluated using electrochemical impedance spectroscopy (EIS). Generally, Warburg impedance is related to diffusion resistance. The Warburg factor (σ) has a linear relation with *Z*′ and is inversely proportional to the diffusion coefficient of electrolyte ions:^56^
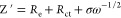
2where *R*_e_ is the electrode-to-electrolyte resistance, *R*_ct_ is the charge transfer resistance, and ω is the angular frequency. As shown in Figure 2h, the slope of *Z*′ against ω^–1/2^ for VO_2_–V_2_O_5_/NC (22.8) is smaller than those of VO_2_/NC (24.5) and V_2_O_5_/NC (31.4), suggesting a larger K^+^ diffusion coefficient and faster diffusion efficiency in the VO_2_–V_2_O_5_/NC electrode.^57^ In addition, the diffusion kinetics of K^+^ in three electrodes were also elucidated by the galvanostatic intermittent titration (GITT) technique. As displayed in Figure S15b, similar trends are found for VO_2_/NC, V_2_O_5_/NC, and VO_2_–V_2_O_5_/NC; however, the K^+^ diffusion coefficient of VO_2_–V_2_O_5_/NC is higher than those of V_2_O_5_/NC and VO_2_/NC, which is consistent with the EIS result.

On the basis of the aforementioned considerations, the outstanding K^+^ storage performance of the VO_2_–V_2_O_5_/NC electrode can be attributed to the following synergistic merits. First, ∼5 nm VO_2_–V_2_O_5_ heterocrystals anchored on a 3D carbon framework expose a large surface area and thus abundant active sites for sufficient electrochemical reactions. Second, compared with single-phase vanadium oxide (VO_2_ or V_2_O_5_), the work function difference between VO_2_ and V_2_O_5_ in VO_2_–V_2_O_5_ heterojunctions will induce the generation of electric fields at the heterointerface,^58^ which can facilitate reaction dynamics and electron/ion transport and strengthen electrochemical performance.^38,59^ Third, ultrasmall VO_2_–V_2_O_5_ heterocrystals on high-conductive carbon sheets not only facilitate electron transfer and high utilization of vanadium oxides but also efficiently buffer their swelling and shrinking and mitigate the mechanical stress during the charge–discharge process.^60^ Fourth, the 3D porous structure of VO_2_–V_2_O_5_/NC benefits the diffusion of electrolyte into pores as well as electron transport throughout the entire composite framework.

### Investigation of K^+^ Storage Mechanism

**Figure 3:**
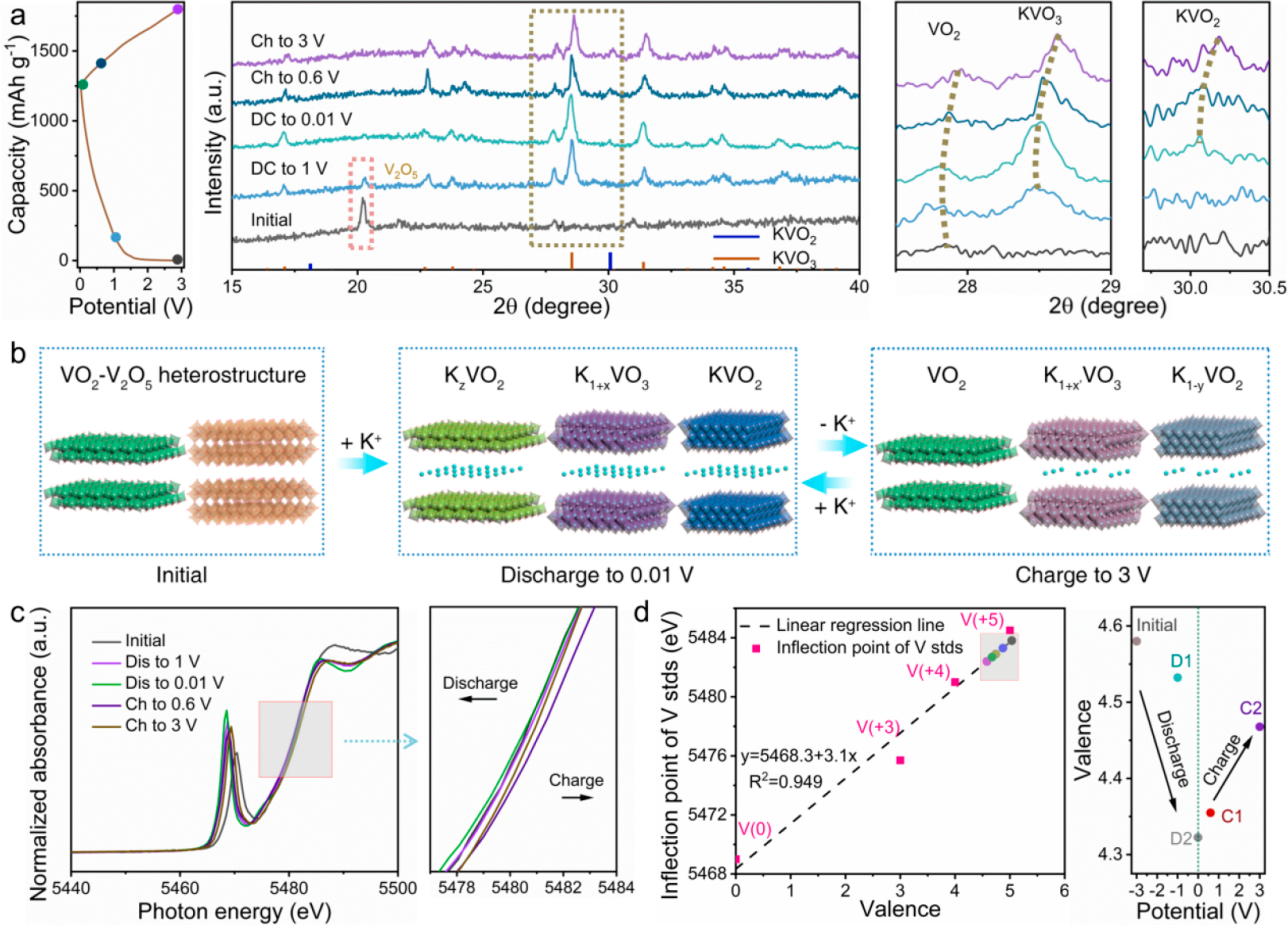
K^+^ storage mechanism of VO_2_–V_2_O_5_/NC electrode. (a) Galvanostatic charge–discharge (GCD) curve in the first cycle (left) and XRD patterns (right) of the VO_2_–V_2_O_5_/NC electrode at different voltage states. (b) Schematic diagram of the potassium storage mechanism of VO_2_–V_2_O_5_/NC, V_2_O_5_/NC, and VO_2_/NC. (c) Normalized vanadium K-edge XANES spectra of the VO_2_–V_2_O_5_/NC electrode discharged to 1 and 0.01 V and then recharged to 0.6 and 3 V in the first cycle. (d) Vanadium oxide-derived linear regressions of vanadium valence at different voltage stages (D1, D2, C1, and C2 indicate the states of VO_2_–V_2_O_5_/NC being discharged to 1, 0.01 V, and charged to 0.6, 3 V, respectively).

To reveal the in-depth K^+^ storage mechanism and understand the local structure evolution, *ex situ* XRD and XAS measurements of VO_2_–V_2_O_5_/NC, V_2_O_5_/NC, and VO_2_/NC electrodes during charge–discharge processes were conducted (Figure 3a, Figure S19). For the V_2_O_5_/NC electrode, V_2_O_5_ transforms into KVO_3_ when discharged to 1 V, and the peaks of KVO_3_ shift slightly to lower angles upon discharging to 0.01 V, indicating the further potassiation and the generation of K_1+*x*_VO_3_. KVO_2_ is also obtained at 0.01 V. During the charging process, K^+^ ions extract from K_1+*x*_VO_3_ and KVO_2_, and V_2_O_5_ partially recovers, resulting in the coexistence of K_1+*x′*_VO_3_ (*x*′ < *x*), K_1–*y*_VO_2_ (*y* < 1), and V_2_O_5_ at 3 V (Figure S19a). On the contrary, only peak shifting occurs in the VO_2_/NC electrode during the charge–discharge processes (Figure S19b), suggesting a reversible K^+^ intercalation/deintercalation energy storage mechanism (VO_2_ ↔ K_*z*_VO_2_). VO_2_–V_2_O_5_/NC inherits the main charge-storage characteristics of both V_2_O_5_/NC and VO_2_/NC, but differences exist (Figure 3b). During the discharge process, K^+^ ions also react with V_2_O_5_ and intercalate into VO_2_, forming K_1+*x*_VO_3_, KVO_2_, and K_*z*_VO_2_, but no V_2_O_5_ is detected after being recharged to 3 V, differing from V_2_O_5_/NC. This significant distinction suggests that there should be some interfacial interactions among the produced K_1+*x*_VO_3_, KVO_2_, and K_*z*_VO_2_ in the VO_2_–V_2_O_5_/NC electrode, which prevent the regeneration of V_2_O_5_ during charging. Notably, the absence of V_2_O_5_ would be capable of reducing phase transition reactions during the following charge–discharge processes, and thus, improved cycle stability for VO_2_–V_2_O_5_/NC is achieved (Figure 2d). Meanwhile, the *ex situ* XRD results also indicate that phase transition reactions and intercalation-induced volume changes should be responsible for the poor cycling stability of the V_2_O_5_/NC and VO_2_/NC electrodes, respectively.^61,62^

The valence shift of vanadium in VO_2_–V_2_O_5_/NC throughout the discharge–charge process was investigated using XANES, which has been shown to be a powerful tool for precisely analyzing transition metal elements (e.g., Mo, Co, Ti).^63−65^Figure 3c (left) shows the normalized vanadium K-edge XANES profiles of VO_2_–V_2_O_5_/NC at selected discharge–charge potentials. During the discharge process, the entire edge position shifted to a lower photon energy level, indicating a decrease of the average vanadium valence state in the VO_2_–V_2_O_5_/NC electrode. In addition, the structural symmetry and geometry of vanadium oxides can be analyzed by the pre-edge peak (∼5470 eV) in XAS spectra. Theoretically, the electrical dipole transition to the p-component in the d–p orbital hybridization order is proportional to the pre-edge peak intensity in 3d transition metal complexes.^50^ With the different electrical distributions in octahedral shape of the d-orbitals, these octahedral vanadium oxides with a 6-coordinated vanadium site have different symmetric distortion orders. Other main feature peaks at 5488 and 5504 eV are attributed to the dipole-allowed 1s–4p transitions from the multiple edge-resonances scattering of the core electrons from higher np states.^66,67^ Herein, the structural symmetry variation of the vanadium site in VO_2_–V_2_O_5_/NC during the discharge–charge cycle can be identified on the basis of these concepts. Figure 3c (right) shows a gradual change in absorbance intensity in the prepeak area, showing that structural symmetry is strengthened and weakened in the discharge and charge processes, respectively, when compared with the pristine condition. With the apparent variations in the structural symmetry and vanadium valence of VO_2_–V_2_O_5_/NC, the occurrences of potassiation and depotassiation are demonstrated. The K^+^ ions in the electrolyte react with vanadium oxides along the crystal planes, thus forming K_1+*x*_VO_3_ and KVO_2_ species with a higher vanadium coordination number and longer V–O bond distance in the duration of discharge.^68^ In the charging process, the entire edge position slightly shifts to higher photon energy, which arises from the partial depotassiation of the vacancy-rich and lattice-distorted K_1+*x*_VO_3_ and KVO_2_, thus reducing back to K_1+*x*′_VO_3_/K_1–*y*_VO_2_ (*x*′ < *x*, *y* < 1) with a higher valence,^35,69^ As depicted in Figure S20, the oscillation amplitude in K^2^-weighted XAS spectra gradually increases with the same frequency during discharging, indicating that the coordination environment of vanadium in the VO_2_–V_2_O_5_/NC electrode varies due to potassiation. Once the charging process occurs, the oscillation amplitude gradually increases, revealing the reduction of vanadium coordination number in the VO_2_–V_2_O_5_/NC electrode toward the original state.

The quantified average valence shift of VO_2_–V_2_O_5_/NC at different voltage stages can be fitted with the vanadium oxide-derived linear regression line (Figure 3d, 1g) with a reliable equation (*y* = 5468.3 + 3.1*x*, *R*^2^ = 0.949). As expected, the average valence state of vanadium in VO_2_–V_2_O_5_/NC decreases during the discharge process. In contrast, the average vanadium valence state increases to a value below the original state during the charging process. This result is in line with K-edge XANES analyses.

### Demonstration of KIC Device Based on VO_2_–V_2_O_5_/NC

**Figure 4:**
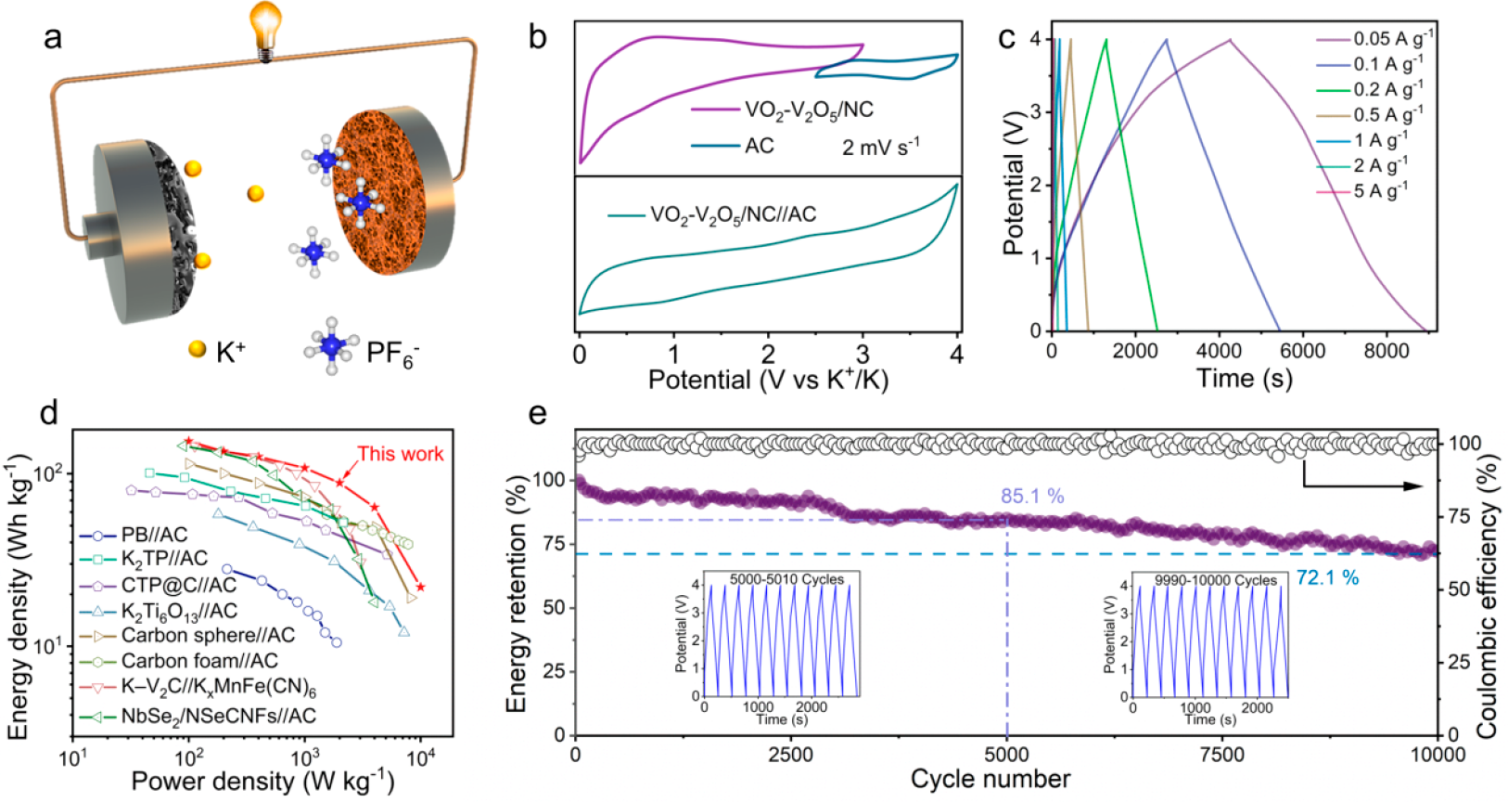
Electrochemical performance of VO_2_–V_2_O_5_/NC//AC KIC. (a) Schematic configuration of the VO_2_–V_2_O_5_/NC//AC KIC device. (b) CV profiles of VO_2_–V_2_O_5_/NC and AC in half cells (top) and VO_2_–V_2_O_5_/NC//AC KIC (bottom). (c) GCD curves of the VO_2_–V_2_O_5_/NC//AC KIC device at different current densities. (d) Ragone plots of VO_2_–V_2_O_5_/NC//AC KIC in comparison with other representative KICs (Prussian blue//AC,^11^ K_2_TP//AC,^12^ Ca_0.5_Ti_2_(PO_4_)_3_@C//AC,^13^ K_2_Ti_6_O_13_//AC,^14^ carbon foam//AC,^15^ K–V_2_C//K_*x*_MnFe(CN)_6_,^16^ NbSe_2_/NSeCNFs//AC^17^). (e) Long cyclic performance of VO_2_–V_2_O_5_/NC//AC KIC at 1 A g^–1^. Insets are the GCD curves from 5000 to 5010, and from 9990 to 10 000 cycles.

To match the performance of the VO_2_–V_2_O_5_/NC anode and construct a high-performance KIC, AC was chosen as the cathodic material (Figure S21a). It shows an working voltage window of 2.5–4 V vs. K^+^/K, a capacity of 39.8 mAh g^–1^ at 0.1 A g^–1^, and an acceptable rate capability (8.3 mAh g^–1^ at 5 A g^–1^) (Figure S21b,c). Moreover, it delivers exceptional cycling stability up to 1200 cycles at 1 A g^–1^ (Figure S21d). The further assembled KICs consisting of VO_2_–V_2_O_5_/NC anode, AC cathode, and KPF_6_ electrolyte are schematically illustrated in Figure 4a. Upon charging, PF_6_^–^ anions are adsorbed to the AC surface, while K^+^ ions are inserted into the VO_2_–V_2_O_5_/NC anode, and these processes reverse during discharge.

Based on the CV profiles of the VO_2_–V_2_O_5_/NC anode and AC cathode, a high and stable operating voltage window up to 4 V was achieved for the constructed VO_2_–V_2_O_5_/NC/AC KIC (Figure 4b, Figure S22). In addition, different anode-to-cathode mass ratios were also examined to maximize the performance of full devices, and the optimal mass ratio of VO_2_–V_2_O_5_/NC anode to AC cathode in our work was determined to be 1:4.5 (Figure S23). Figure 4c presents the GCD curves of VO_2_–V_2_O_5_/NC//AC KIC at different current densities (0.05–5 A g^–1^). All the GCD profiles show high symmetry and deviate from the linear characteristics of ideal supercapacitors, indicating efficient and complex K^+^ storage processes in our device.^6,7^ According to the GCD curves, the maximum gravimetric energy (power) density of the as-fabricated VO_2_–V_2_O_5_/NC//AC KIC reaches 154 Wh kg^–1^ (100 W kg^–1^) (based on the total active mass of two electrodes, a high gravimetric energy density of 22 Wh kg^–1^ still maintains at an ultrahigh power density of 10 000 W kg^–1^; Figure 4d). The energy/power densities of our device outperform the most high-performance KICs ever reported (Table S3). In addition, the VO_2_–V_2_O_5_/NC//AC KIC device also exhibits exceptional cycling stability with high capacity retention of 85.1% after 5000 cycles and 72.1% even after 10 000 cycles (Figure 4e), demonstrating its great potential for mid/large-scale practical applications.

## Conclusion

To conclude, a high-energy and long-term stable K-ion storage anode with ultrasmall VO_2_–V_2_O_5_ nanoheterostructures anchored on 3D porous N-doped carbon networks was successfully developed. Because of the ultrasmall size of VO_2_–V_2_O_5_ heterocrystals, favorable interfacial effect, high-conductive 3D carbon network, and distinctive K-ion storage mechanism, the VO_2_–V_2_O_5_/NC electrode exhibits high K-ion storage performance, such as a high reversible capacity of 510 mAh g^–1^ and significant long-term stability with a capacity of 252 mAh g^–1^ after 1600 cycles. Furthermore, a KIC device consisting of VO_2_–V_2_O_5_/NC anode and commercial AC cathode delivers a high gravimetric energy density of 154 Wh kg^–1^ at 100 W kg^–1^, as well as a long lifespan, retaining 85.1% capacity after 5000 cycles and 72.1% capacity even after 10 000 cycles. This study provides a pathway for designing high-energy and long-term potassium-ion storage electrodes and devices for practical applications.

## Methods

### Preparation of VO_2_–V_2_O_5_/NC

A self-template strategy is employed to synthesize ultrasmall VO_2_–V_2_O_5_ nanoheterojunctions fixed on 3D N-doped carbon network (VO_2_–V_2_O_5_/NC). First, a homogeneous solution was prepared by dissolving 0.36 g of oxalic acid (Alfa Aesar) and 0.23 g of ammonium vanadate (Aladdin) in 80 mL of deionized water. Then 2 g of dicyandiamide (Sigma-Aldrich) was added in and dried at 80 °C. The as-collected precursor was then calcined at 800 °C for 2 h in N_2_ atmosphere with a heating rate of 2 °C min^–1^ to produce the VN/NC composite. After that, the as-obtained VN/NC was reheated to 280 °C in air at a heating rate of 1°C min^–1^ and maintained for 4 h, VO_2_–V_2_O_5_/NC was finally obtained. For comparison, VO_2_/*g*-C_3_N_4_ was prepared by annealing the ammonium vanadate/dicyandiamide precursor at 550 °C for 2 h in a N_2_ atmosphere at a heating rate of 2 °C min^–1^. N-doped carbon (NC) was prepared by calcining an oxalic acid (0.1 g)/dicyandiamide (1 g) mixture at 800 °C for 2 h in a N_2_ atmosphere at a heating rate of 2 °C min^–1^. For comparison, VO_2_/NC was prepared by heating VN/NC at 260 °C in air for 6 h with a heating rate of 1 °C min^–1^, while V_2_O_5_/NC was formed by annealing VN/NC at 280 °C in air for 8 h.

## Supporting Information Available

Methods of material characterizations and electrochemical measurements; SEM, TEM, TGA, XRD, XPS, N_2_ adsorption/desorption isotherms, and BET surface areas; additional electrochemical characterizations and performance; and additional references (PDF)
