# Supplementary material for: Interfacial Engineered Vanadium Oxide Nanoheterostructures Synchronizing High-Energy and Long-Term Potassium-Ion Storage
Source: ACS Nano. 2022 Jan 11;16(1):1502–10. doi: 10.1021/acsnano.1c09935 (PMC8793133; doi:10.1021/acsnano.1c09935)
Supplement: Supplementary file 1 — nn1c09935_si_001.pdf [file nn1c09935_si_001.pdf]

Supporting information

Interfacial Engineered Vanadium Oxide Nanoheterostructures Synchronizing High-Energy and Long-Term Potassium-Ion Storage

Xiaoxiao Kuai,[†,∇] Ke Li,[‡,∇] Jianmei Chen,[ω,∇] Hao Wang,[δ,*] Junyi Yao,[†] Chao-Lung Chiang,[ξ] Tingting Liu,[ψ] Hanzhang Ye,[ψ] Jianqing Zhao,[†] Yan-Gu Lin,[ξ] Labao Zhang,[δ] Valeria Nicolosi,[‡,*] and Lijun Gao[†,*]

[†]Soochow Institute for Energy and Materials Innovations & Key Laboratory of Advanced Carbon Materials and Wearable Energy Technologies of Jiangsu Province, College of Energy, Soochow University, Suzhou 215006, China

[‡]School of Chemistry, Centre for Research on Adaptive Nanostructures and Nanodevices (CRANN) & Advanced Materials and BioEngineering Research (AMBER), Trinity College Dublin, Dublin, Dublin 2, Ireland

[ω]College of Electronic and Optical Engineering & College of Microelectronics, Nanjing University of Posts and Telecommunications, Nanjing, 210023, P. R. China

[δ]Research Institute of Superconductor Electronics, School of Electronic Science and Engineering, Nanjing University, Nanjing 210023, China

[ξ]National Synchrotron Radiation Research Center, Hsinchu 30076, Taiwan, R.O.C.

[ψ]School of Environmental Science and Engineering& Jiangsu Key Laboratory of Environmental Science and Engineering, Suzhou University of Science and Technology, Suzhou 215001, China

Corresponding authors:

Dr. Hao Wang, Email: wanghao91@nju.edu.cn

Prof. Valeria Nicolosi, Email: nicolov@tcd.ie

Prof. Lijun Gao, Email: gaolijun@suda.edu.cn

▽These authors contributed equally to this work.

Experimental Section

Material characterization.

Scanning electron microscopy (SEM) images were taken on FEI Quanta 250 FEG instrument at an accelerating voltage of 20 kV. Transmission electron microscopy (TEM), high-resolution TEM (HRTEM), high-angle annular dark-field scanning transmission electron microscopy (HAADF-STEM) and corresponding energy dispersive spectroscopy (EDS) mapping analysis were carried out on the FEI Tecnai G-20 microscope, equipped with a field-emission gun that operated at 200 kV. X-ray diffraction (XRD) patterns were recorded on a Bruker D8 Advance Diffractometer X-ray diffractometer. X-ray photoelectron spectroscopy (XPS) was performed on a spectrometer from Thermal Fisher, using a single Al Ka (1486 eV) light source. The energy level was calibrated to the C 1s peak maximum at 284.8 eV. The different voltage states of VO_2-V_2O_5/NC samples and standards were analyzed by using X-ray absorption near-edge structure spectra (XANES) collected at Taiwan Photon Source Beamline (TPS 44A) station of the National Synchrotron Radiation Research Center

(NSRRC) in Hsinchu, Taiwan. The operation energy and ring current of electron storage ring were respectively 3.0 GeV and 500 mA, supporting the operation of TPS 44A station. In the energy range of vanadium K-edge (5460 eV), a Lytle detector was used to collect the XANES spectra at 25 ˚C. During data processing in Athena software (version 0.9.26), the photon energy of these collected XANES spectra were first calibrated with the highest inflection point in the first derivative XANES spectra of vanadium standard foil at 5460 eV. The positions of the highest inflection point in the XANES spectrum of the first derivative of the sample were double-confirmed, and the corresponding points in the XANES spectrum of the second derivative of the sample were a zero crossing. Afterward, these data processed XANES spectra were normalized for further comparisons. The wavevector K^2-weighted and R-space of samples were Fourier-transformed (FT) from these calibrated and normalized extended X-ray absorption fine structure (EXAFS) spectra to clarify the effects of K^+ insertion/extraction on surrounding central vanadium atoms.

Electrochemical measurements.

The electrochemical evaluation was performed on 2032-type coin cells. To make the working electrode, VO_2-V_2O_5/NC, Super P, and polyvinylidene difluoride (PVDF) binder were mixed in N-methyl pyrrolidone (NMP) with a weight ratio of 80:10:10. Slurry was cast onto copper foil using a doctor-blade technique with the areal mass loading of active materials is 3-4 mg cm^{-2}. The film was dried in a vacuum oven at 110 ˚C for 12 h. For K half-cell, the reference and counter electrode were K metal foil,

the electrolyte was a 0.8 M KPF_6 in a mixture of ethylene carbonate, dimethyl carbonate, and diethyl carbonate (1:1:1 by volume), and cells were assembled using Whatman GF/A glass-fiber fitters as separators. The K-ion batteries were assembled in a glove box full of argon, oxygen, and moisture concentration is less than 0.5 ppm. Galvanostatic charge-discharge (GCD) tests were performed on a LAND CT2001A battery test system in the voltage range of 0.01-3 V. Cyclic voltammetry (CV) profiles were recorded on an AUTOLAB PGSTAT302N electrochemical workstation at scan rates from 0.1 to 500 mV s^{-1} in a potential range of 0.01-3 V. The full-battery was constructed with a VO_2-V_2O_5/NC anode and active carbon cathode with a weight ratio of 1:4.5. The active carbon, Super P, and PVDF binder were mixed in NMP with a weight ratio of 90:5:5 onto aluminum foils. The pre-assembly VO_2-V_2O_5/NC electrode was first cycled pre-filled in a semi-battery, using a low current density of 0.05 A g^{-1} until the Coulomb efficiency exceeds 95%, and then the VO_2-V_2O_5/NC electrode was transferred to the full battery for testing. The current density was based on the total mass of positive and negative active materials. Using the following formula, the constant current discharge profile was numerically integrated to calculate the ratio energy and ratio power of the entire battery:

$$E = \int_{t1}^{t2} iV/m \, dt = \Delta V \times \frac{i}{m} \times t, \tag{3}$$

$$P = \frac{E}{t}, \tag{4}$$

where i is charge and discharge current (A), V is the working voltage (V), t1 and t2 are beginning and ending time (s) of discharge, m is the weight of active materials of

both positive and negative electrodes.

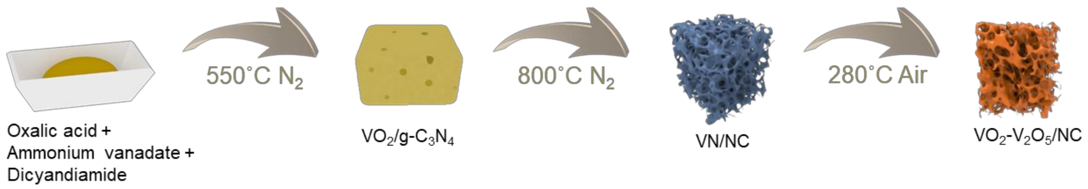

Figure S1. Schematic illustration of synthesis of VO_2-V_2O_5/NC.

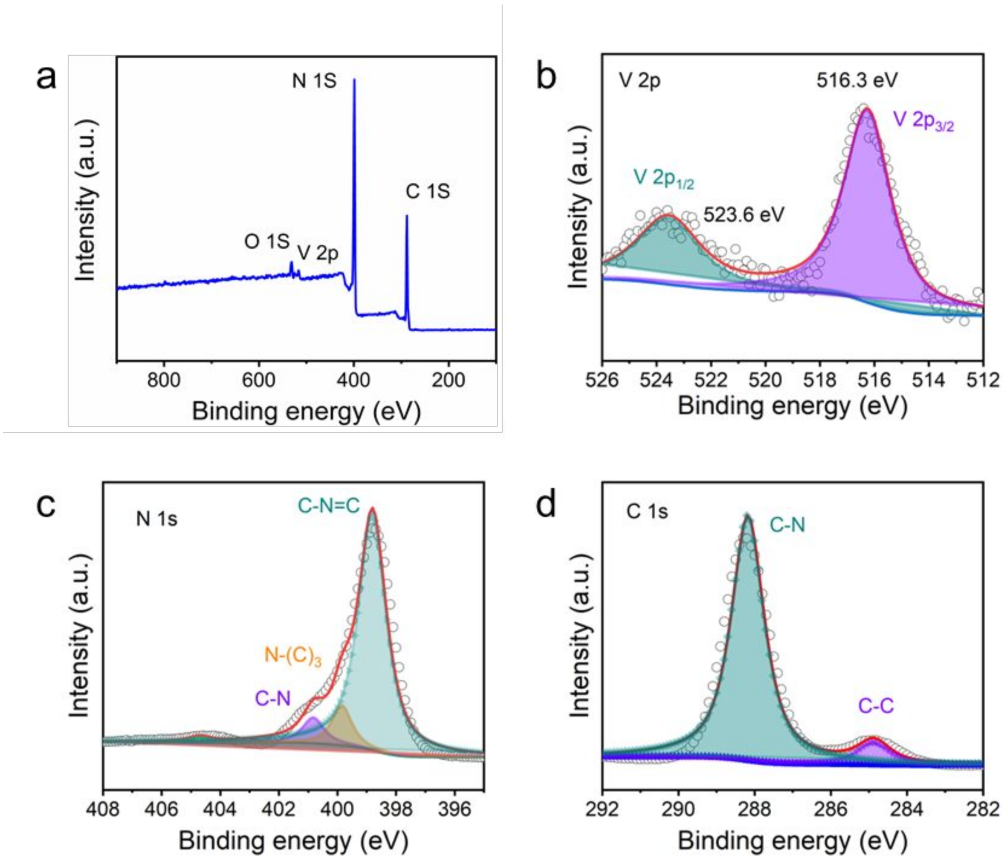

Figure S2. XPS analysis of VO_2/*g*-C_3N_4. (a) XPS full scan and high-resolution XPS spectra of (b) V 2p, (c) N 1s, and (d) C 1s. Peaks around 516.3 and 523.6 eV correspond to V $2p_{3/2}$ and $2p_{1/2}$.

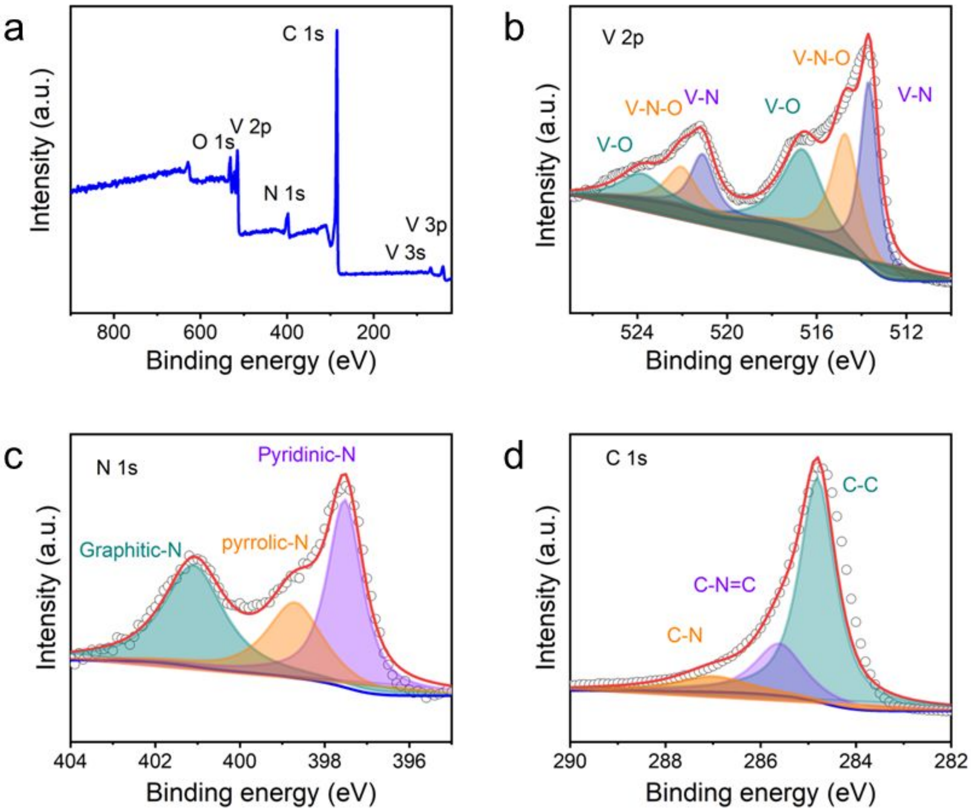

Figure S3. XPS analysis of VN/NC. (a) XPS full scan and high-resolution XPS spectra of (b) V 2p, (c) N 1s and (d) C 1s. Duo to the slight oxidation of VN, peaks around 513.7 and 523.3 eV can be divided to V-N, V-N-O and V-O, respectively.

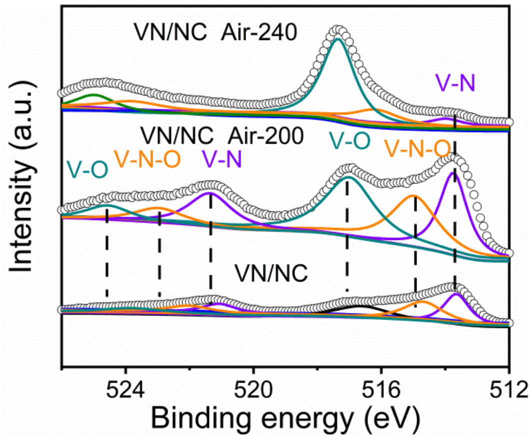

Figure S4. High-resolution XPS spectra of V in VN/NC, VN/NC annealed at 200 ˚C in air and VN/NC annealed at 240 ˚C in air.

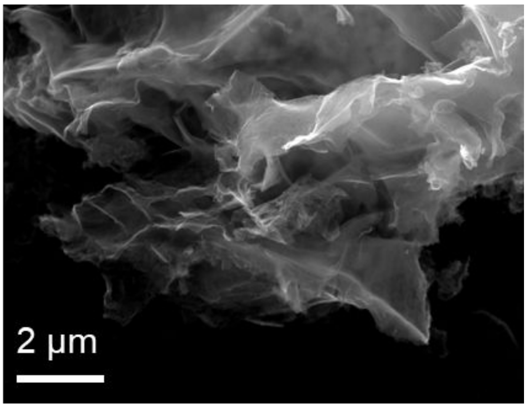

Figure S5. SEM image of 3D VN/NC.

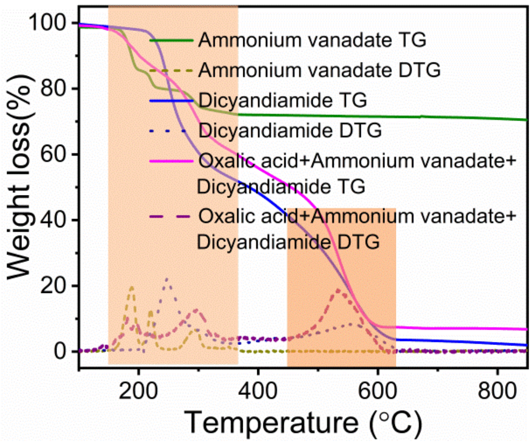

Figure S6. TG/DTG curves of ammonium vanadate, dicyandiamide and oxalic acid/ammonium vanadate/dicyandiamide in N_2 atmosphere. Ammonium vanadate followed a classic three-stage decomposition and was fully decomposed into V_2O_5 when being heated at temperatures above 370 ^{o}C. In previous report,[1] the dicyandiamide was transformed into g-C_3N_4 at ~530 ^{o}C, and being fully decomposed at 700 ^{o}C. Referring to the oxalic acid/ammonium vanadate/dicyandiamide mixture, similar to dicyandiamide, VO_2/g-C_3N_4 was formed at ~530 ^{o}C. Due to the interaction between the lone-pair electrons in pyridinic N and $(VO)_2(C_2O_4)_3^{2-}$, g-C_3N_4 were not fully decomposed but being carbonized into 3D N-doped carbon network at higher temperature, which supported the nanosized VN.

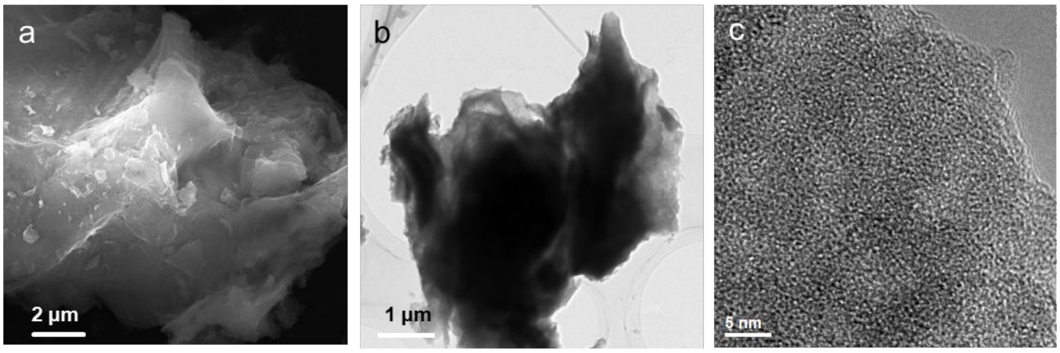

Figure S7. Morphology of $VO_2/g\text{-}C_3N_4$. SEM (a), TEM (b) and HRTEM (c) images reveal the bulky structure of $VO_2/g\text{-}C_3N_4$.

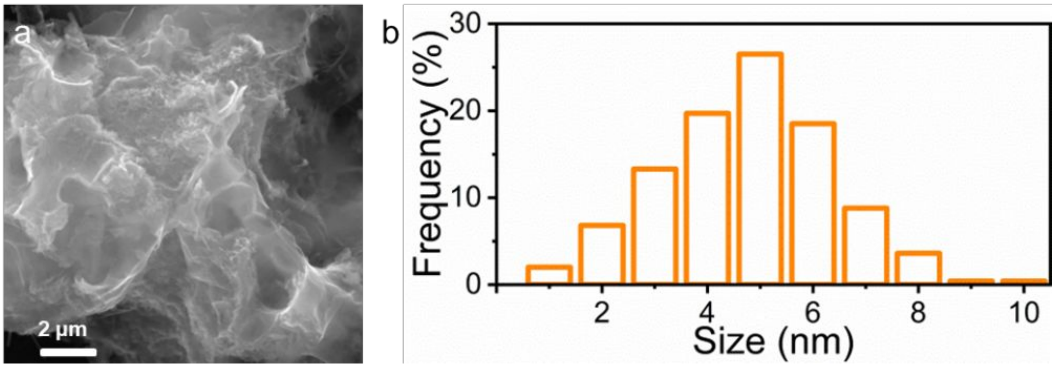

Figure S8. (a) SEM image reveals the porous structure of VO_2-V_2O_5/NC. (b) Size distribution of VO_2-V_2O_5/NC nanocrystals.

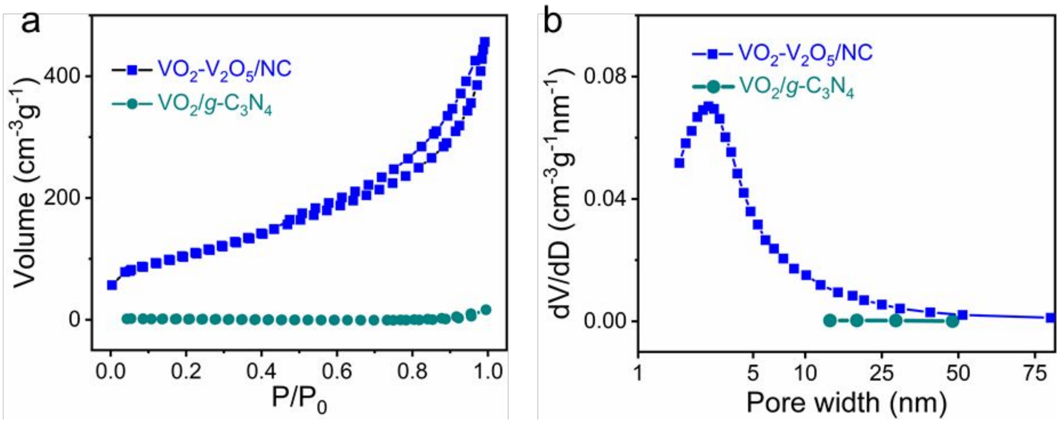

Figure S9. (a) N_2 adsorption/desorption isotherms of VO_2-V_2O_5/NC and VO_2/*g*-C_3N_4. (b) Corresponding pore size distribution curves. A type-II isotherm with a hysteresis loop indicates mesoporous structure.[2]

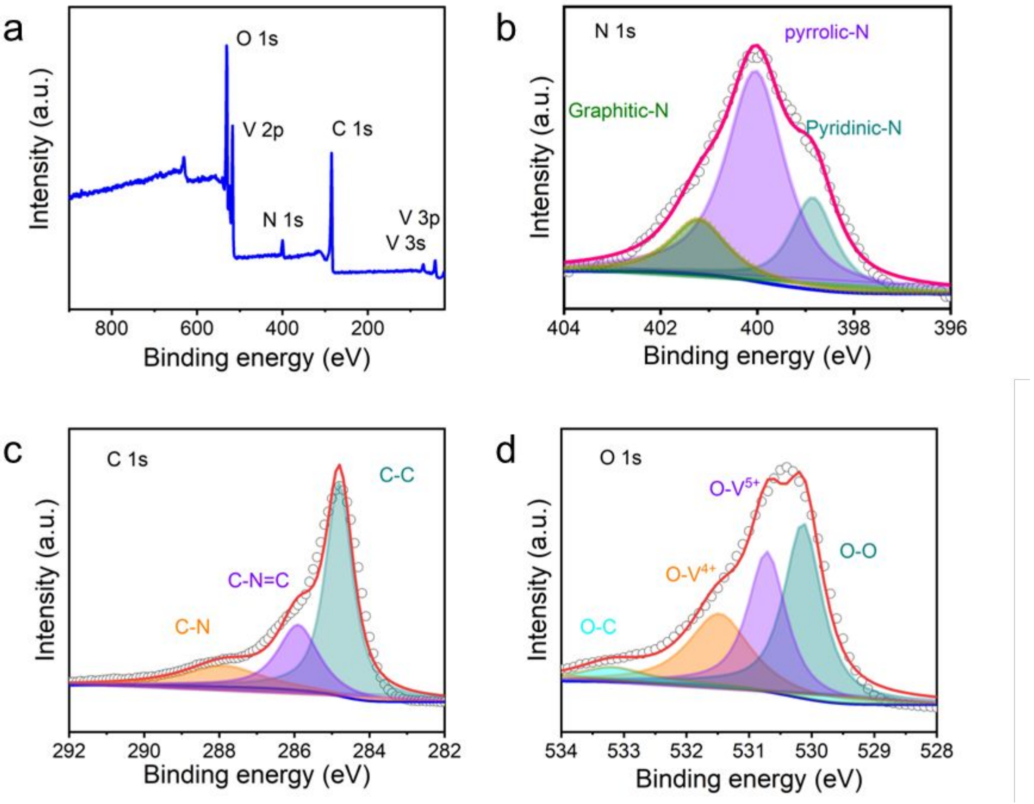

Figure S10. XPS analysis of VO_2-V_2O_5/NC. (a) XPS full scan and high-resolution XPS spectra of (b) N 1s, (c) C 1s and (d) O 1s. The high-resolution XPS spectrum of N 1s can be deconvolved into 3 peaks, including graphitic N (401.2 eV), pyrrolic N

(400 eV) and pyridinic N (398.8 eV), which confirm the successful N-doping in carbon network.[3] The high-resolution XPS of C1s further confirms the N-doping in carbon network, and the high-resolution XPS of O 1s confirms the existence of V^{4+} and V^{5+}.

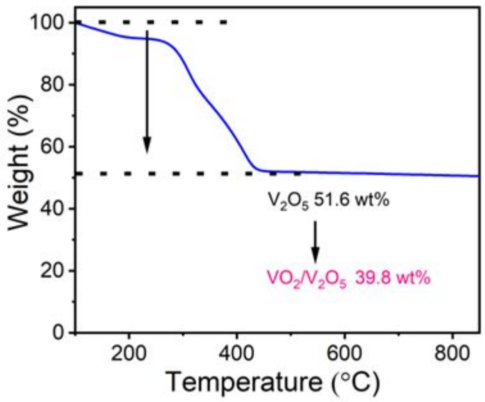

Figure S11. TGA curve of VO_2-V_2O_5/NC in air with a heating rate of 5 °C min^{-1}. The weight loss at around 430 °C corresponds to the removal of carbon. As approved by XRD and XPS that, VO_2-V_2O_5/NC was consisting of 42 wt% VO_2 and 58 wt% V_2O_5 ($0.42 * 4 + 0.58 * 5 = 4.58$). Assuming the VO_2 were completely conversed into V_2O_5 during the test, the weight percentage of VO_2-V_2O_5 was calculated to be 39.8 wt%.

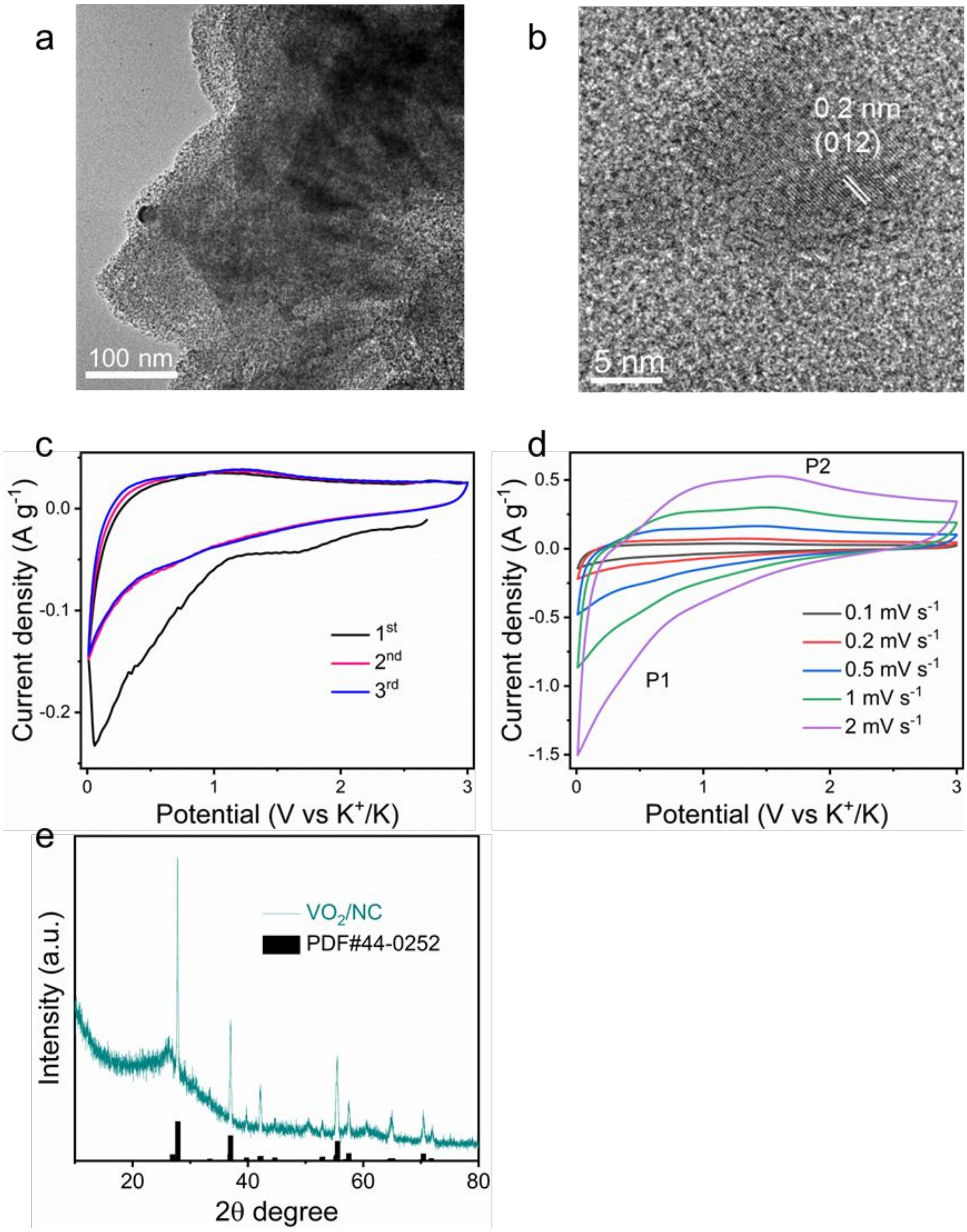

Figure S12. Morphological and CV profiles of VO_2/NC. (a) TEM and (b) HRTEM images of VO_2/NC. (c) The first three CV cycles of VO_2/NC at 0.1 mV s^{-1}. (d) CV profiles of VO_2/NC at scan rates of 0.1-2 mV s^{-1}. (e) XRD pattern of VO_2/NC.

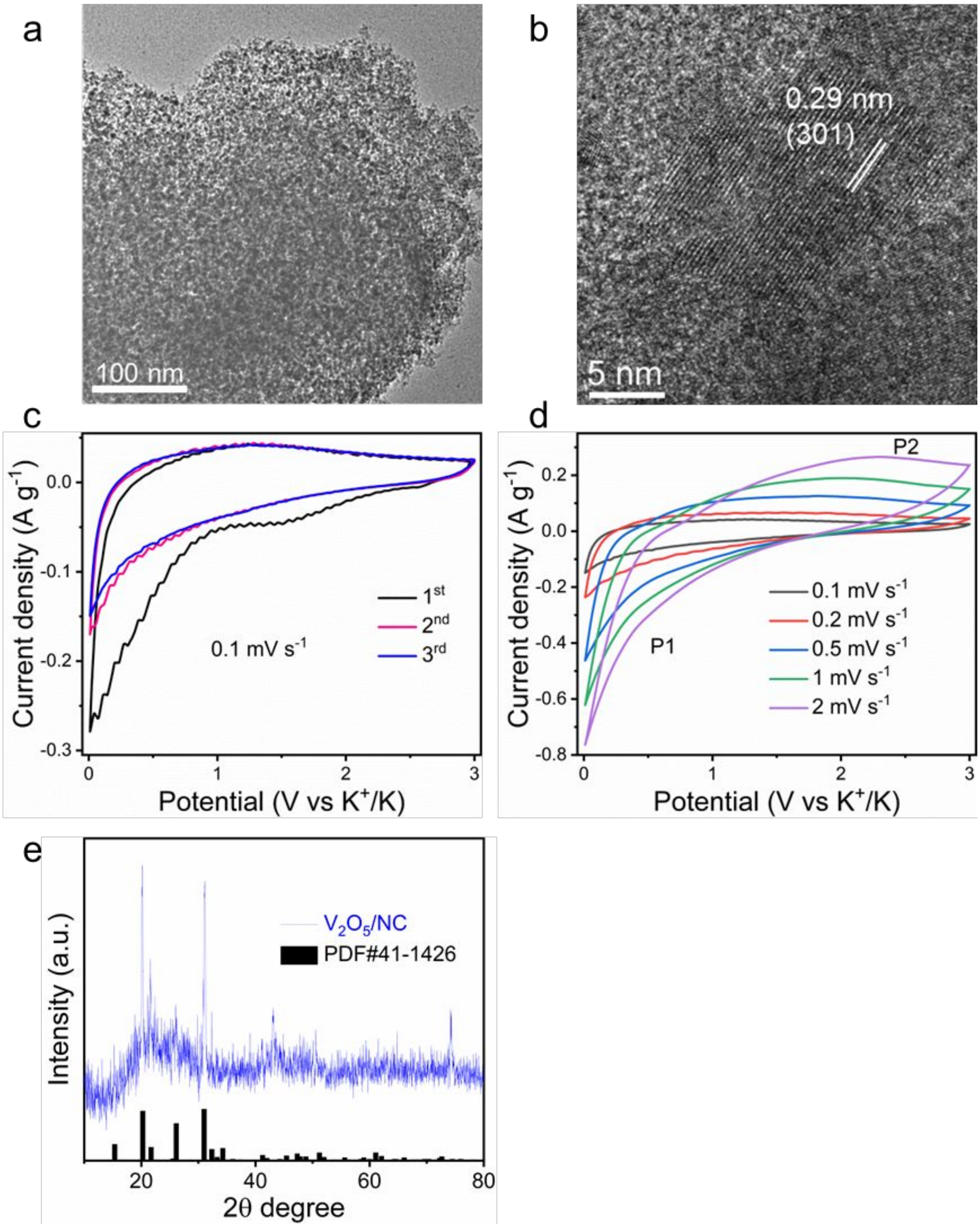

Figure S13. Morphological and CV profiles of V_2O_5/NC. (a) TEM and (b) HRTEM images of V_2O_5/NC. (c) The first three CV cycles of V_2O_5/NC at 0.1 mV s^{-1}. (d) CV profiles of V_2O_5/NC at scan rates of 0.1-2 mV s^{-1}. (e) XRD pattern of V_2O_5/NC.

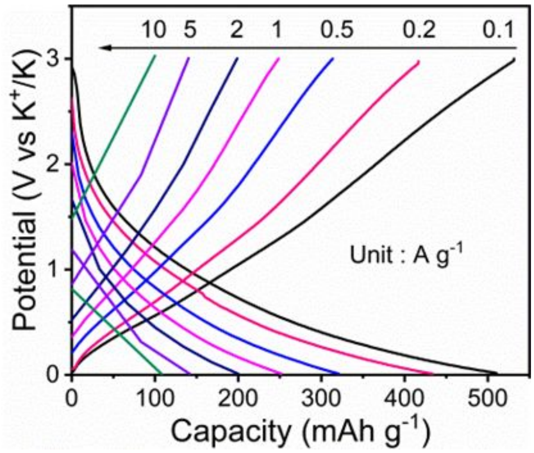

Figure S14. The charge-discharge curves of VO_2-V_2O_5/NC at different current densities.

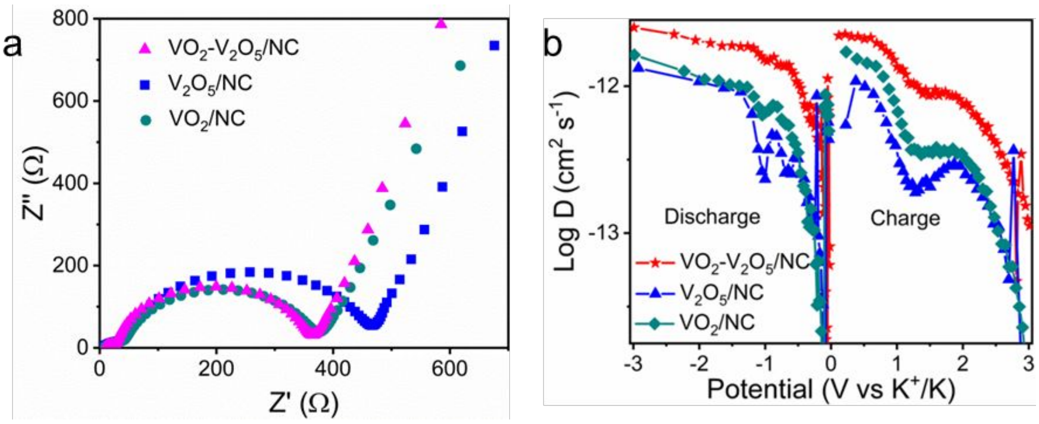

Figure S15. (a) EIS data of VO_2-V_2O_5/NC, VO_2/NC, and V_2O_5/NC. (b) The corresponding K^+ diffusion coefficient of VO_2-V_2O_5/NC, V_2O_5/NC and VO_2/NC electrodes.

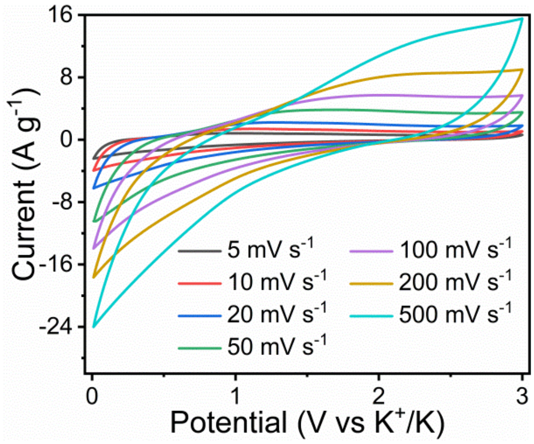

Figure S16. CV profiles of VO_2-V_2O_5/NC at scan rates of 5-500 mV s^{-1}.

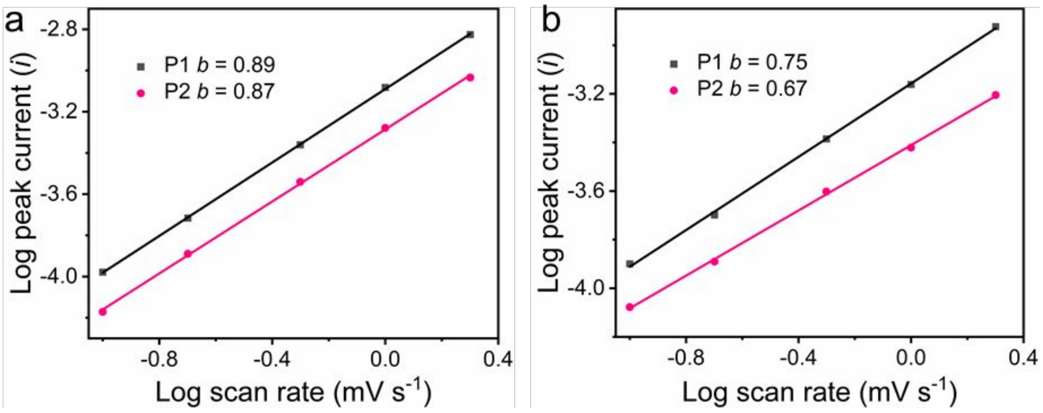

Figure S17. *b*-value determination according to the relationship between peak current i and scan rate v, $i = av^b$, log i *vs*. log v relationship.[4] (a) VO_2/NC, (b) V_2O_5/NC.

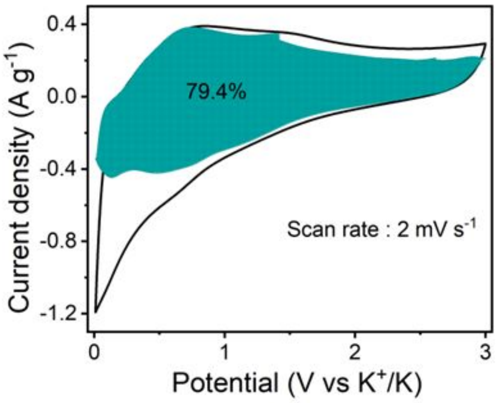

Figure S18. CV curves of VO_2-V_2O_5/NC electrode with separation between total and surface-capacitive current densities at 2 mV s^{-1}.

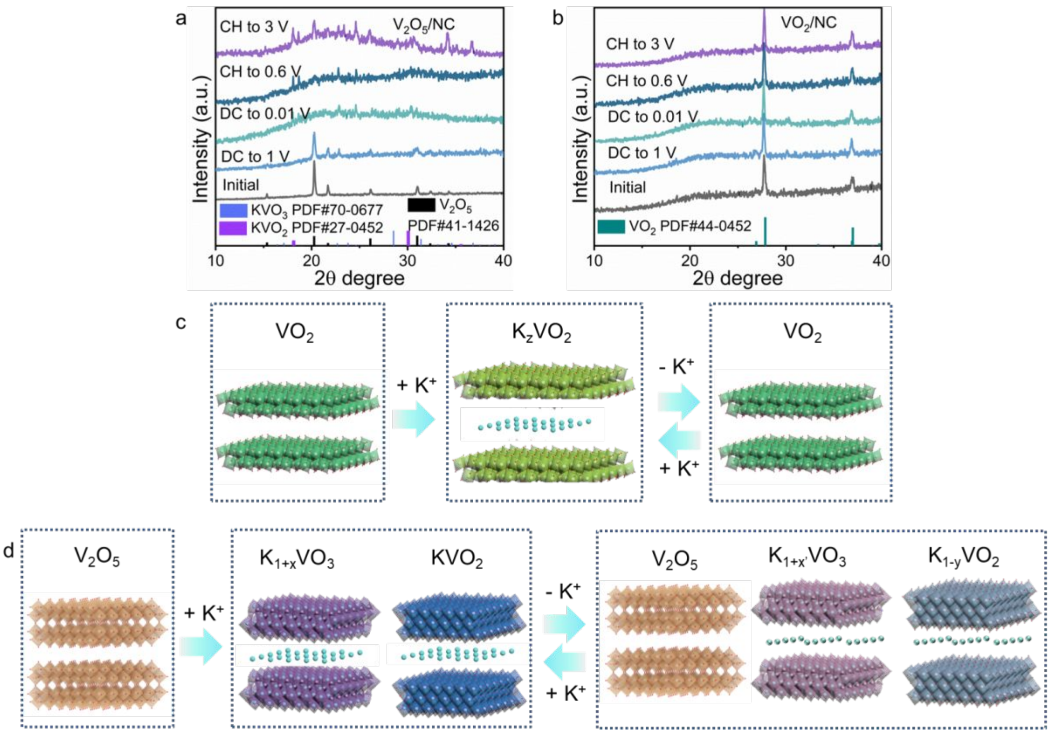

Figure S19. XRD patterns of (a) V_2O_5/NC and (b) VO_2/NC electrodes at different voltage states. Schematic diagram of potassium storage mechanism of (c) VO_2/NC, and (d) V_2O_5/NC.

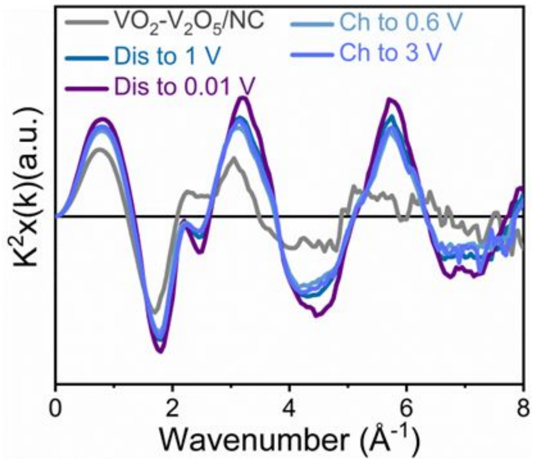

Figure S20. The k^2-weighted spectra of VO_2-V_2O_5/NC electrode.

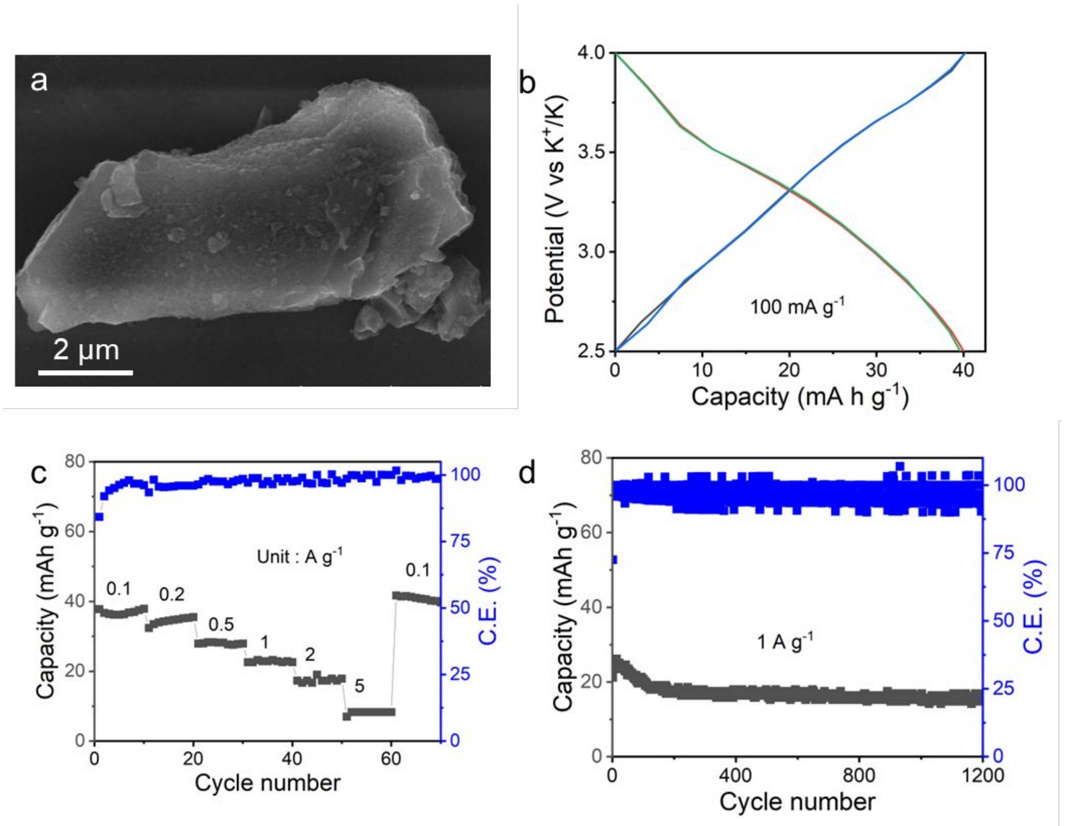

Figure S21. SEM image and electrochemical properties of AC cathode. (a) SEM image of AC. (b) GCD profiles of AC at 100 mA g^{-1}. (c) Rate capacity of AC cathode. (d) Long-life cycling performance of AC cathode at 1 A g^{-1}.

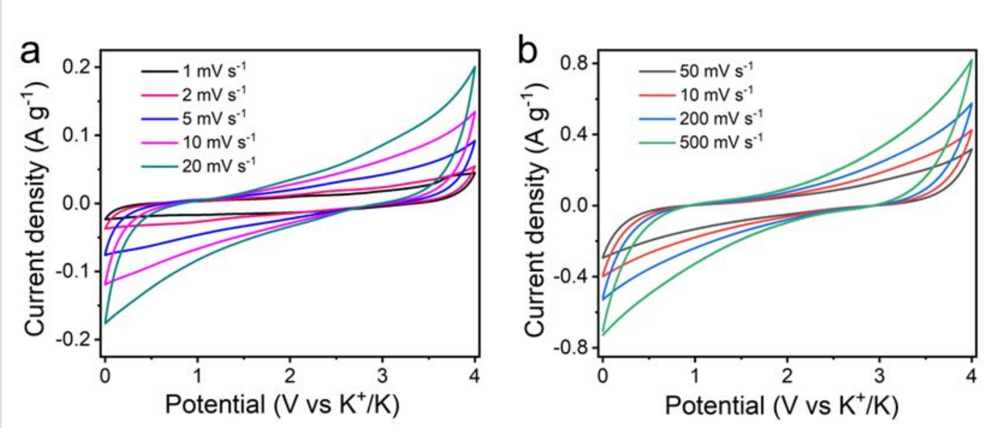

Figure S22. CV profiles of the VO_2-V_2O_5/NC//AC KIC device in a potential range of 0-4 V at scan rates of 1-500 mV s^{-1}.

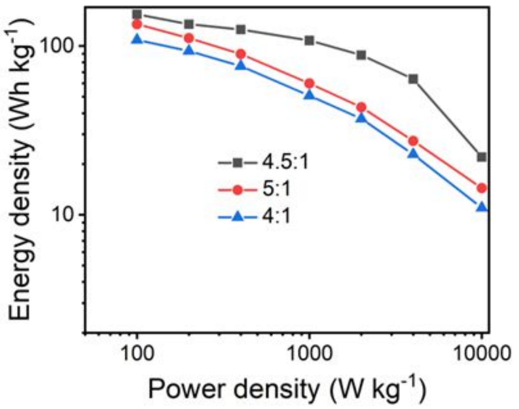

Figure S23. Ragone plots of KIC devices with different active cathode: active anode ratios.

Table S1

	Surface area (m^{2} g^{-1})	Pore volume (cm^{3} g^{-1})	Average pore width (nm)
VO_2-V_2O_5/NC	378.5	1.385	6.3
VO_2/g-C_3N_4	2.1	0.051	47.9

Table S2. Electrochemical performance of VO_2-V_2O_5/NC-based K-ion batteries (KIB) and others.

Systems	Potential window (V)	Long cycle performance (mAh g^{-1})	Rate capacity (mAh g^{-1})	Carbon content (%)	Mass loading (mg cm^{-2})	Ref.
**VO_2-V_2O_5/NC K-ion battery**	**0.01-3**	**252 mAh g^{-1} at 1 A g^{-1} (1600 cycles)**	**501 mAh g^{-1} at 0.1 A g^{-1} 108 mAh g^{-1} at 10 A g^{-1}**	**60.2**	**3-4**	**This work**
SA-VO_2 K-ion battery	0.1-3	177.1 mAh g^{-1} at 0.5 A g^{-1} (500 cycles)	290.2 mAh g^{-1} at 0.05A g^{-1} 141.4 mAh g^{-1} at 2 A g^{-1}	0	~0.8	5

VO_2/carbon foam K-ion battery	0.01-3	236.4 mAh g^{-1} at 1 A g^{-1} (400 cycles)	443.7 mAh g^{-1} at 0.1 A g^{-1} 251.7 mAh g^{-1} at 2 A g^{-1}	60.38	NA	6
V_2O_3@C K-ion battery	0.01-3	147.9 mAh g^{-1} at 2 A g^{-1} (1800 cycles)	217.4 mAh g^{-1} at 0.05 A g^{-1} 116.6 mAh g^{-1} at 5 A g^{-1}	25.7	0.8-1.2	7
V_2O_3@PNC NFs K-ion battery	0.01-3	229.9 mAh g^{-1} at 0.05 A g^{-1} (500 cycles)	240 mAh g^{-1} at 0.05 A g^{-1} 134 mAh g^{-1} at 1 A g^{-1}	40.3	NA	8
K-VO_2(B) K-ion battery	0.01-3	122 mAh g^{-1} at 1 A g^{-1} (500 cycles)	310 mAh g^{-1} at 0.2 A g^{-1} 151 mAh g^{-1} at 2 A g^{-1}	0	NA	9
VN-QDs/CM-600 K-ion battery	0.01-3	215 mAh g^{-1} at 0.5 A g^{-1} (500 cycles)	261 mAh g^{-1} at 0.1 A g^{-1} 152 mAh g^{-1} at 2 A g^{-1}	~13.8	~1	10
K-V_2C K-ion battery	0.01-3	NA	195 mAh g^{-1} at 0.05 A g^{-1} 70 mAh g^{-1} at 3 A g^{-1}	0	1.5-2	11
$Ca_{0.5}Ti_2(PO_4)_3$@C K-ion battery	0.01-3	74.6 % at 1A g^{-1} (1000 cycles)	224 mAh g^{-1} at 0.05 A g^{-1} 63 mAh g^{-1} at 5 A g^{-1}	19.3	~1	12
$K_2Ti_6O_{13}$ K-ion battery	0.1-2.5	59 mAh g^{-1} at 0.5 A g^{-1} (1000 cycles)	95 mAh g^{-1} at 0.02 A g^{-1} 64 mAh g^{-1} at 0.5 A g^{-1}	0	NA	13
N, S-3DHPC-600 K-ion battery	0.01-3	249.5 mAh g^{-1} at 1 A g^{-1} (1000 cycles)	380.5 mAh g^{-1} at 0.1 A g^{-1} 129.4 mAh g^{-1} at 10 A g^{-1}	100	0.8	14
$NbSe_2$/NSeC NFs//AC K-ion battery	0.01-3	NA	NA	36	1.5	15
Carbon sphere K-ion battery	0.01-3	~104.8 mAh g^{-1} at 5.6 A g^{-1} (500 cycles)	280 mAh g^{-1} at 0.028 A g^{-1} 129.4 mAh g^{-1} at 5.6 A g^{-1}	100	NA	16
Carbon foam K-ion battery	1.5-4.8	123.4 mAh g^{-1} at 1 A g^{-1} (500 cycles)	149.6 mAh g^{-1} at 1 A g^{-1} 107.1 mAh g^{-1} at 5 A g^{-1}	100	NA	17
Carbon foam	0.01-3	226.3 mAh	NA	100	NA	17

K-ion battery		g^{-1} at 1 A g^{-1} (500 cycles)				
3D CFM-SNG K-ion battery	0.01-2.5	188.8 mAh g^{-1} at 1 A g^{-1} (2000 cycles)	348.2 mAh g^{-1} at 0.05 A g^{-1}<br>204.3 mAh g^{-1} at 2 A g^{-1}	100	NA	18
HNCNS K-ion battery	0.05-3	296 mAh g^{-1} at 0.1 A g^{-1} (400 cycles)	343 mAh g^{-1} at 0.1 A g^{-1}<br>91 mAh g^{-1} at 3 A g^{-1}	100	NA	19
Dipotassium terephthalate //AC K-ion battery	0.2-1.4	220 mAh g^{-1} at 0.5 A g^{-1} (100 cycles)	270 mAh g^{-1} at 0.1 A g^{-1}<br>126 mAh g^{-1} at 5 A g^{-1}	0	NA	20
3D-NTC750 K-ion battery	0.01-3	~246.7 mAh g^{-1} at 1 A g^{-1} (500 cycles)	518 mAh g^{-1} at 0.05 A g^{-1}<br>119 mAh g^{-1} at 5 A g^{-1}	100	1	21
CNTs/GCF K-ion battery	0.01-2.5	226 mAh g^{-1} at 0.1 A g^{-1} (800 cycles)	254 mAh g^{-1} at 0.05 A g^{-1}<br>74 mAh g^{-1} at 1 A g^{-1}	100	0.5-1	22
$Fe_{x-1}Se_x$/MXene/Carbonaceous K-ion battery	0.01-3	449.3 mAh g^{-1} at 0.1 A g^{-1} (80 cycles)<br>254.3 mAh g^{-1} at 1 A g^{-1} (900 cycles)	479.6 mAh g^{-1} at 0.1 A g^{-1}<br>55.3 mAh g^{-1} at 2 A g^{-1}	~45	1.3-1.5	23
$K_{0.22}V_{1.74}O_{4.37}$ • 0.82 H_2O Aqueous K-ion battery in 1M KCl	-0.1-0.9	NA	183 mAh g^{-1} at 5 mV s^{-1}<br>93 mAh g^{-1} at 200 mV s^{-1}	0	~1.93	24
$K_{0.22}V_{1.74}O_{4.37}$ • 0.82 H_2O Aqueous K-ion battery in 3M KCl	-0.6-0.6	62.5 mAh g^{-1} at 2 A g^{-1} (5000 cycles)<br>~50 mAh g^{-1} at 5 A g^{-1} (5000 cycles)<br>~45 mAh g^{-1} at 10 A g^{-1} (5000 cycles)<br>40 mAh g^{-1} at 20 A g^{-1} (5000 cycles)	NA	0	~1.93	24
Cubic	0-1.1	80 mAh g^{-1} at	80 mAh g^{-1} at 0.5 A	0	1.5	25

Prussian blue Aqueous K-ion battery in 0.5 M K_2SO_4		0.5 A g^{-1} (After rate rest, 800 cycles)	g^{-1} 26 mAh g^{-1} at 5 A g^{-1}			

Table S3. Electrochemical performance of VO_2-V_2O_5/NC//AC KIC and other KICs or KIB full cells.

Systems	Potential window / V	Maximum energy density / Wh kg^{-1}	Maximum power density / W kg^{-1}	Ref
**VO_2-V_2O_5/NC//AC KIC**	**0-4**	**154 Wh kg^{-1}/100 W kg^{-1}**	**22 Wh kg^{-1}/10000 W kg^{-1}**	**This work**
K-V_2C//$K_xMnFe(CN)_6$ KIC	0-4.6	145 Wh kg^{-1}/112.6 W kg^{-1}	30.55 Wh kg^{-1}/3026 W kg^{-1}	11
$Ca_{0.5}Ti_2(PO_4)_3$@C //AC KIC	1-4	80 Wh kg^{-1}/32 W kg^{-1}	34 Wh kg^{-1}/5144 W kg^{-1}	12
$K_2Ti_6O_{13}$//NGC KIC	0-3.5	58.2 Wh kg^{-1}/175 W kg^{-1}	12 Wh kg^{-1}/7200 W kg^{-1}	13
N, S-3DHPC-600//AC-800 KIC	0.01-4.2	130.6 Wh kg^{-1}/210 W kg^{-1}	56 Wh kg^{-1}/16800 W kg^{-1}	14
$NbSe_2$/NSeCNFs//AC KIC	0.01-3.8	145 Wh kg^{-1}/90 W kg^{-1}	18 Wh kg^{-1}/4000 W kg^{-1}	15
Carbon sphere//AC KIC	0.01-4	100.5 Wh kg^{-1}/114.2 W kg^{-1}	19.1 Wh kg^{-1}/8203 W kg^{-1}	16
Carbon foam KIC	0.01-3.8	58 Wh kg^{-1}/1558.2W kg^{-1}	39 Wh kg^{-1}/7800 W kg^{-1}	17
Dipotassium terephthalate //AC KIC	0.1-3.2	101 Wh kg^{-1}/46W kg^{-1}	52 Wh kg^{-1}/2160W kg^{-1}	20
3D-NTC750//PTCDA KIB Full cell	0.5-3.5	187 Wh kg^{-1}/NA	NA	21
Cubic Prussian blue//AC KIC	0-1.6	28 Wh kg^{-1}/214W kg^{-1}	10.5 Wh kg^{-1}/1890W kg^{-1}	23
$Fe_{x-1}Se_x$/MXene/Carbonaceous//$K_3V_2(PO_4)_3$	0.4-3.9	NA	NA	25

References

(1) Li, D. J.; Maiti, U. N.; Lim, J.; Choi, D. S.; Lee, W. J.; Oh, Y.; Lee, G. Y.; Kim,

S. O. Molybdenum Sulfide/N-doped CNT Forest Hybrid Catalysts for High-Performance Hydrogen Evolution Reaction. *Nano Lett.* **2014**, 14 (3), 1228-1233.

(2) Su, J.; Yang, Y.; Xia, G.; Chen, J.; Jiang, P.; Chen, Q. Ruthenium-Cobalt Nanoalloys Encapsulated in Nitrogen-Doped Graphene as Active Electrocatalysts for Producing Hydrogen in Alkaline Media. *Nat. Commun.* **2017**, 8 (1), 1-12.

(3) Chen, Y.-Y.; Zhang, Y.; Jiang, W.-J.; Zhang, X.; Dai, Z.; Wan, L.-J.; Hu, J.-S. Pomegranate-Like N, P-doped Mo_2C@C Nanospheres as Highly Active Electrocatalysts for Alkaline Hydrogen Evolution. *ACS Nano* **2016**, 10 (9), 8851-8860.

(4) Augustyn, V.; Come, J.; Lowe, M. A.; Kim, J. W.; Taberna, P.-L.; Tolbert, S. H.; Abruña, H. D.; Simon, P.; Dunn, B. High-Rate Electrochemical Energy Storage through Li^+ Intercalation Pseudocapacitance. *Nat. Mater.* **2013**, 12 (6), 518-522.

(5) Li, Y.; Zhang, Q.; Yuan, Y.; Liu, H.; Yang, C.; Lin, Z.; Lu, J. Surface Amorphization of Vanadium Dioxide (B) for K-Ion Battery. *Adv. Energy Mater.* **2020**, 10 (23), 2000717.

(6) Jin, D.; Gao, Y.; Zhang, D.; Wei, Y.; Chen, G.; Qiu, H.; Meng, X. VO_2@Carbon Foam as a Freestanding Anode Material for Potassium-Ion Batteries: First Principles and Experimental Study. *J. Alloys Compd.* **2020**, 845, 156232.

(7) Hu, J.; Xie, Y.; Zheng, J.; Li, H.; Wang, T.; Lai, Y.; Zhang, Z. Encapsulating V_2O_3 Nanoparticles in Hierarchical Porous Carbon Nanosheets *via* C–O–V Bonds for Fast and Durable Potassium-Ion Storage. *ACS Appl. Mater. Interfaces* **2021**, 13 (10),

12149-12158.

(8) Jin, T.; Li, H.; Li, Y.; Jiao, L.; Chen, J. Intercalation Pseudocapacitance in Flexible and Self-Standing V_2O_3 Porous Nanofibers for High-Rate and Ultra-Stable K Ion Storage. *Nano Energy* **2018**, 50, 462-467.

(9) Li, Q.; Ye, X.; Jiang, Y.; Ang, E. H.; Liu, W.; Feng, Y.; Rui, X.; Yu, Y. Superior Potassium and Zinc Storage in K-Doped VO_2(B) Spheres. *Mater. Chem. Front.* **2021**, 5 (7), 3132-3138.

(10) Wu, H.; Yu, Q.; Lao, C.-Y.; Qin, M.; Wang, W. A.; Liu, Z.; Man, C.; Wang, L.; Jia, B.; Qu, X. Scalable Synthesis of VN Quantum Dots Encapsulated in Ultralarge Pillared N-Doped Mesoporous Carbon Microsheets for Superior Potassium Storage. *Energy Stor. Mater.* **2019**, 18, 43-50.

(11) Ming, F.; Liang, H.; Zhang, W.; Ming, J.; Lei, Y.; Emwas, A.-H.; Alshareef, H. N. Porous MXenes Enable High Performance Potassium Ion Capacitors. *Nano Energy* **2019**, 62, 853-860.

(12) Zhang, Z.; Li, M.; Gao, Y.; Wei, Z.; Zhang, M.; Wang, C.; Zeng, Y.; Zou, B.; Chen, G.; Du, F. Fast Potassium Storage in Hierarchical $Ca_{0.5}Ti_2(PO_4)_3$@C Microspheres Enabling High-Performance Potassium-Ion Capacitors. *Adv. Funct. Mater.* **2018**, 28 (36), 1802684.

(13) Dong, S.; Li, Z.; Xing, Z.; Wu, X.; Ji, X.; Zhang, X. Novel Potassium-Ion Hybrid Capacitor Based on an Anode of $K_2Ti_6O_{13}$ Microscaffolds. *ACS Appl. Mater. Interfaces* **2018**, 10 (18), 15542-15547.

(14) Fan, B.; Yan, J.; Hu, A.; Liu, Z.; Li, W.; Li, Y.; Xu, Y.; Zhang, Y.; Tang, Q.;

Chen, X. High-Performance Potassium Ion Capacitors Enabled by Hierarchical Porous, Large Interlayer Spacing, Active Site Rich-Nitrogen, and Sulfur Co-Doped Carbon. *Carbon* **2020**, 164, 1-11.

(15) Chen, M.; Wang, L.; Sheng, X.; Wang, T.; Zhou, J.; Li, S.; Shen, X.; Zhang, M.; Zhang, Q.; Yu, X. An Ultrastable Nonaqueous Potassium-Ion Hybrid Capacitor. *Adv. Funct. Mater.* **2020**, 30 (40), 2004247.

(16) Qiu, D.; Guan, J.; Li, M.; Kang, C.; Wei, J.; Li, Y.; Xie, Z.; Wang, F.; Yang, R. Kinetics Enhanced Nitrogen-Doped Hierarchical Porous Hollow Carbon Spheres Boosting Advanced Potassium-Ion Hybrid Capacitors. *Adv. Funct. Mater.* **2019**, 29 (32), 1903496.

(17) Feng, Y.; Chen, S.; Wang, J.; Lu, B. Carbon Foam with Microporous Structure for High Performance Symmetric Potassium Dual-Ion Capacitor. *J. Energy Chem.* **2020**, 43, 129-138.

(18) Yang, W.; Zhou, J.; Wang, S.; Wang, Z.; Lv, F.; Zhang, W.; Zhang, W.; Sun, Q.; Guo, S. A Three-Dimensional Carbon Framework Constructed by N/S Co-Doped Graphene Nanosheets with Expanded Interlayer Spacing Facilitates Potassium Ion Storage. *ACS Energy Lett.* **2020**, 5 (5), 1653-1661.

(19) Kim, S.; Ju, M.; Lee, J.; Hwang, J.; Lee, J. Polymer Interfacial Self-Assembly Guided Two-Dimensional Engineering of Hierarchically Porous Carbon Nanosheets. *J. Am. Chem. Soc.* **2020**, 142 (20), 9250-9257.

(20) Luo, Y.; Liu, L.; Lei, K.; Shi, J.; Xu, G.; Li, F.; Chen, J. A Nonaqueous Potassium-Ion Hybrid Capacitor Enabled by Two-Dimensional Diffusion Pathways of

Dipotassium Terephthalate. *Chem. Sci.* **2019**, 10 (7), 2048-2052.

(21) Zhang, W.; Yin, J.; Sun, M.; Wang, W.; Chen, C.; Altunkaya, M.; Emwas, A. H.; Han, Y.; Schwingenschlögl, U.; Alshareef, H. N. Direct Pyrolysis of Supermolecules: An Ultrahigh Edge-Nitrogen Doping Strategy of Carbon Anodes for Potassium-Ion Batteries. *Adv. Mater.* **2020**, 2000732.

(22) Zeng, S.; Zhou, X.; Wang, B.; Feng, Y.; Xu, R.; Zhang, H.; Peng, S.; Yu, Y. Freestanding CNT-Modified Graphitic Carbon Foam as a Flexible Anode for Potassium Ion Batteries. *J. Mater. Chem. A* **2019**, 7 (26), 15774-15781.

(23) Zhou, L.; Zhang, M.; Wang, Y.; Zhu, Y.; Fu, L.; Liu, X.; Wu, Y.; Huang, W. Cubic Prussian Blue Crystals from a Facile One-Step Synthesis as Positive Electrode Material for Superior Potassium-Ion Capacitors. *Electrochim. Acta* **2017**, 232, 106-113.

(24) Charles, D. S.; Feygenson, M.; Page, K.; Neuefeind, J.; Xu, W.; Teng, X. Structural Water Engaged Disordered Vanadium Oxide Nanosheets for High Capacity Aqueous Potassium-Ion Storage. *Nat. Commun.* **2017**, 8 (1), 15520.

(25) Cao, J.; Wang, L.; Li, D.; Yuan, Z.; Xu, H.; Li, J.; Chen, R.; Shulga, V.; Shen, G.; Han, W. $Ti_3C_2T_x$ MXene Conductive Layers Supported Bio-Derived $Fe_{x-1}Se_x$/MXene/Carbonaceous Nanoribbons for High-Performance Half/Full Sodium-Ion and Potassium-Ion Batteries. *Adv. Mater.* **2021**, 33, 2101535.
